# Broadband measurement of true transverse relaxation rates in systems with coupled protons: application to the study of conformational exchange†

**DOI:** 10.1039/d1sc03391c

**Published:** 2021-08-03

**Authors:** Peter Kiraly, Guilherme Dal Poggetto, Laura Castañar, Mathias Nilsson, Andrea Deák, Gareth A. Morris

**Affiliations:** Department of Chemistry, University of Manchester Oxford Road Manchester M13 9PL UK g.a.morris@manchester.ac.uk; Eötvös Loránd Research Network (ELKH), Research Centre for Natural Sciences, Institute of Materials and Environmental Chemistry, Supramolecular Chemistry Research Group Magyar Tudósok körútja 2 1117 Budapest Hungary

## Abstract

Accurate measurement of transverse relaxation rates in coupled spin systems is important in the study of molecular dynamics, but is severely complicated by the signal modulations caused by scalar couplings in spin echo experiments. The most widely used experiments for measuring transverse relaxation in coupled systems, CPMG and PROJECT, can suppress such modulations, but they also both suppress some relaxation contributions, and average relaxation rates between coupled spins. Here we introduce a new experiment which for the first time allows accurate broadband measurement of transverse relaxation rates of coupled protons, and hence the determination of exchange rate constants in slow exchange from relaxation measurements. The problems encountered with existing methods are illustrated, and the use of the new method is demonstrated for the classic case of hindered amide rotation and for the more challenging problem of exchange between helical enantiomers of a gold(i) complex.

## Abstract 2

Existing methods for measuring transverse relaxation give incorrect results in coupled spin systems. Measuring true relaxation rates extends their utility.

## Graphical abstract

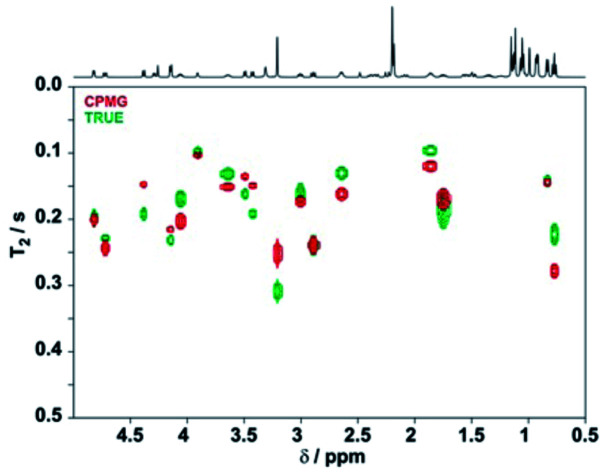

## 1 Introduction

The spin echo is a vital tool in modern pulsed NMR experiments, suppressing the effects of chemical shift and static field inhomogeneity on NMR signals. In principle this allows *inter alia* measurement of the rates of decoherence – transverse relaxation – of NMR signals. Such relaxation both limits linewidths, and carries information about molecular motion and structure and about the rates of chemical processes.^1,2^ In all but the simplest NMR experiments it also interferes with accurate quantitation. However, the experimental methods that are commonly used for measuring transverse relaxation do not work reliably in coupled spin systems. Here the reasons for this are explained and their practical consequences illustrated, and a general new method is described that allows the true transverse relaxation rates of all individual coupled spins in a system to be measured simultaneously. The utility of the new method is illustrated with measurements of slow exchange rates in two systems, a classic example of hindered amide rotation in diethylformamide and the more complicated case of interconversion between enantiomeric conformers of the gold complex [Au_2_(μ-xantphos)_2_](NO_3_)_2_.

Measurements of transverse relaxation are used in a broad range of chemical and biochemical applications, including studies of the dynamics of molecules, chemical exchange, detection of NMR-invisible excited states using relaxation dispersion, and ligand binding studies. For example, the measurement of ^1^H transverse relaxation of isolated methyl groups (*i.e.* those with no scalar couplings) has been applied to study methylation processes in DNA samples.^3^ In biomolecular applications, it is the transverse relaxation of the sparse ^15^N nuclei, for which homonuclear coupling can be neglected, that is most often studied.^4^ Such relaxation dispersion experiments are growing in popularity, but their recent applications to ^1^H NMR are restricted to the small minority of protons that have negligible homonuclear couplings.^5^ An innovative recent example is the use of selective ^13^CHD_2_ labelling of amino acids in proteins,^6^ allowing characterisation of faster motions using ^1^H and ^13^C relaxation dispersion experiments.^7^ The scope of such experiments would be significantly expanded if the complications caused by the presence of homonuclear couplings could be avoided.

**Fig. 1:**
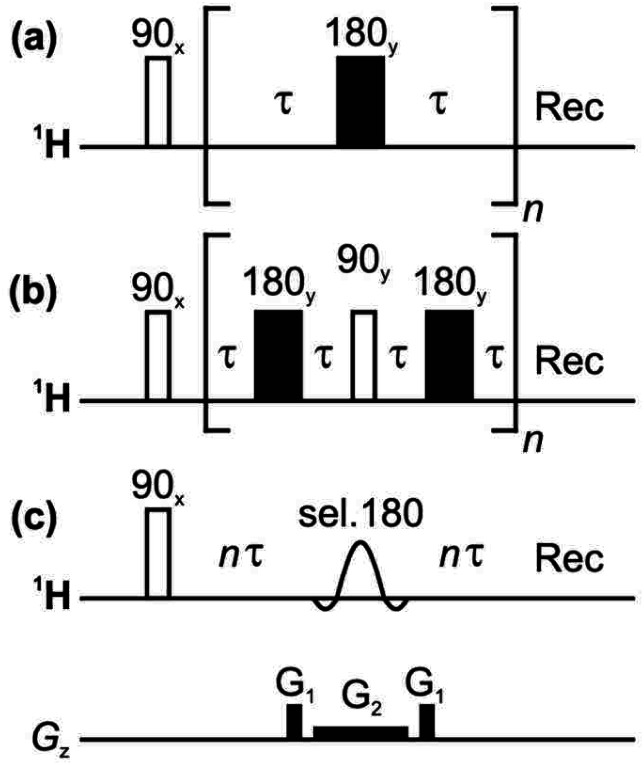
Pulse sequences for measuring *T*_2_ relaxation: (a) CPMG, (b) PROJECT, and (c) TRUE using a Zangger-Sterk (ZS) element. The decay of the NMR signal is measured as a function of *n* in each case.

The primary problem with using spin echoes to measure transverse relaxation is *J* modulation, which causes the phases of multiplet components to vary periodically. Transverse relaxation is usually assumed to be exponential with a time constant *T*_2_, although in coupled spin systems this is only an approximation. *T*_2_ measurements have until recently usually been performed using the Carr–Purcell–Meiboom–Gill (CPMG) experiment^8–10^ (Fig. 1a); if used with rapid refocusing (short delays *τ*) this quenches *J* modulation, albeit at the cost of sample heating. The more recent PROJECT (Periodic Refocusing of *J* Evolution by Coherence Transfer) method^11^ (Fig. 1b) greatly reduces the rate of refocusing that is needed to suppress *J* modulation, avoiding excessive sample heating, by using the ‘perfect echo’ pulse sequence element.^12,13^

Unfortunately, although the nonselective CPMG experiment has been used for measurements of *T*_2_ values for protons in coupled spin systems, neither it nor PROJECT gives a true measure of the transverse relaxation of individual protons in such systems. CPMG and PROJECT share two fundamental problems: first, both of these experiments suppress some important sources of transverse relaxation; and second, both of them cause exchange of transverse magnetization between coupled spins. In the limit of fast refocusing, the latter exchange of magnetization corresponds to isotropic mixing in CPMG, and to planar mixing in PROJECT (see Section 4.1). One consequence, as shown below, is that if a rapidly-relaxing spin is coupled to a slowly-relaxing one, the apparent *T*_2_ measured for the rapidly-relaxing spin by CPMG or PROJECT can be greater than its spin-lattice relaxation time, *T*_1_.

Still further complications arise in coupled spin systems because of the contribution made to the transverse relaxation of a given spin by the longitudinal relaxation of spins that are coupled to it.^14–16^ In proton NMR this is usually a significant, and sometimes the dominant, contribution to linewidths in complex multiplets. Taking the simplest example of a two spin-½ AX spin system, the scalar coupling *J*_AX_ leads to a spin A doublet in which one component derives from molecules in which spin X is in the α state and the other from molecules with X in the β state. Spin-lattice relaxation of spin X interconverts the α and β states of X, in turn interconverting the magnetizations of the two doublet components of A. The rate constant for this process is 1/(2*T*_1_^X^); just as with chemical exchange, if 1/(2*T*_1_^X^) < ∼*J*_AX_ it leads to line broadening, and if 1/(2*T*_1_^X^) ≫ *J*_AX_ to the collapse of the A spin doublet to a singlet. At intermediate values of 1/(2*T*_1_^X^), oscillations are seen in the signal decay, and the individual lines in multiplets are no longer pure absorption mode Lorentzians (see ESI† section 1). One largely unremarked consequence of this is that experimental spectra of coupled spin systems show small systematic deviations from the spectra simulated with standard software packages. In the AX case the effect of spin-lattice relaxation of spin X can be regarded as a mixture of “in-phase relaxation” and “antiphase” relaxation.^17–19^ In the slow limit the effect of the longitudinal relaxation of spin X is to increase the average rate of transverse relaxation of spin A by 1/(2*T*_1_^X^). This effect can be regarded as a third form of scalar relaxation, distinct from the “scalar relaxation of the first kind” and “scalar relaxation of the second kind” of Abragam;^20^ for simplicity, it will simply be referred to as “scalar relaxation” in what follows. In more complex spin systems each resolved coupling *J*_i_ will make its own contribution 1/(2*T*_1_^i^).

An important secondary reason for making transverse relaxation measurements (and the initial stimulus for the present work) is to allow multiple pulse NMR experiments to be used for quantitation. Any experiment in which the time between signal excitation and measurement is not negligible compared to *T*_2_ will yield signal integrals that depend on *T*_2_. This is a significant limitation, for example, on quantitative use of pure shift NMR methods.^21,22^ By definition, such experiments are intended for use in coupled spin systems, so to be able to correct the signal amplitude distortions introduced by the different transverse relaxation rates of different spins we need a relaxation measurement method that is not, like CPMG and PROJECT, confounded by exchange of magnetization (and hence averaging of the effects of relaxation) between coupled spins. A further requirement is that the measurement method should reflect transverse relaxation during free precession, *i.e.* relaxation in the absence of radiofrequency irradiation. Any method such as CPMG, PROJECT, or spin locking, that applies repeated pulses or continuous irradiation, will suppress contributions to transverse relaxation from low frequency spectral density (*e.g.* from chemical exchange or slow motion), increasing the measured *T*_2_; indeed this is the basis of CPMG relaxation dispersion measurements.^3,4,6,7^

At the risk of courting controversy, methods such as CPMG and PROJECT, despite their popularity, do not measure the “true” *T*_2_, *i.e.* the rate of spin decoherence unperturbed by radiofrequency irradiation. Historically, the only practical way to determine true *T*_2_ values in coupled spin systems has been to measure individual spins only, by the use of line- or multiplet-selective refocusing pulses or spin locking.^16,23^ Here we describe for the first time a simple approach that allows transverse relaxation to be measured for all coupled spins in a spectrum simultaneously. This is achieved by borrowing from ‘pure shift’ NMR methods^21,22^ the technique of selecting a sparse subset of spins to be observed, and refocusing these individual spins without perturbing the spins to which they are coupled, thus avoiding *J* modulation. A schematic pulse sequence diagram for the new experiment, TRUE (Transverse Relaxation Unmodulated Echo) is shown in Fig. 1c. In the example illustrated, the Zangger-Sterk (ZS) pulse sequence element is used for the selective refocusing;^24^ alternatives include BIRD^25,26^ and PSYCHE.^27^ The ZS element consists of a selective 180° pulse in the presence of a magnetic field gradient, and restricts the spins observed to a different horizontal slice of the NMR sample for each chemical shift. Only these spins are inverted, leaving the remaining spins unaffected. In pure shift NMR the ZS element is used in combination with a nonselective 180° pulse, to refocus couplings but not chemical shifts; here the ZS element is used on its own to refocus both shifts and couplings, suppressing *J* modulation without perturbing transverse relaxation with extra radiofrequency pulses. The TRUE *T*_2_ experiment is thus a multiplexed analogue of *T*_2_ measurement using multiplet-selective 180° pulses,^16^ the field gradient pulse allowing spin-selective echoes to be recorded for all signals in parallel. The price paid for this broadband character is a significant reduction in signal-to-noise ratio.

We first illustrate the importance of the often-neglected phenomenon of scalar relaxation, using numerical simulations of *T*_2_ experiments performed using Spinach.^28^ Next, experimental results for different *T*_2_ measurement methods applied to cyclosporin and azithromycin are compared. Finally, we illustrate the use of the new TRUE method to measure rate constants in slow exchange, and hence determine activation energies, for two dynamic systems. In the case of classic hindered rotation in *N*,*N*-diethylacetamide the methyl multiplets are resolved but the methylene overlap, so Hoffman–Forsén (selective inversion recovery) measurements are only possible for the former but TRUE can be applied to both. In the interconversion of helical enantiomers in the complex [Au_2_(μ-xantphos)_2_](NO_3_)_2_, which features an aurophilic interaction,^29,30^ Hoffman–Forsén measurements give misleading results at low temperatures because of intermethyl cross-relaxation.

## 2 Simulations

Numerical simulations of conventional ^1^H NMR spectra and of CPMG, PROJECT and TRUE experiments were carried out using version 1.8 of the Spinach software package,^28^ in order to illustrate the effects of relaxation in a simple two-spin system AX without the complications introduced by experimental imperfections such as field inhomogeneity. A chemical shift difference of 500 Hz was used with the coupling *J*_AX_ set either to 0 or 10 Hz, so that strong coupling effects were minimal. The spin-lattice relaxation times *T*_1_ were set to 0.2 and 0.8 s respectively for A and X, with the intrinsic spin–spin relaxation time (*i.e.* that in the absence of coupling effects) for each spin *T*_2_ = *T*_1_. The relaxation model used in Spinach was “T1T2”.^28^ Delays *τ* of 200 and 800 μs were used for CPMG and PROJECT respectively, as typical values used in these experiments. The TRUE experiment was simulated using an 18.5 ms RSNOB selective pulse, corresponding to 100 Hz effective bandwidth. The actual (as opposed to intrinsic) *T*_2_ values were determined from the widths at half-height of the peaks in the simulated spectra. Apparent *T*_2_ values were determined by exponential fitting of the results of simulations of CPMG, PROJECT and TRUE simulations using total relaxation-encoding times of 6.4, 12.8, 25.6, 51.2, 102.4, 204.8, 409.6, and 819.2 ms.

## 3 Experimental

NMR experiments were carried out on 500 MHz Varian/Agilent VNMRS, 500 MHz Bruker Neo, and 400 MHz Varian Inova spectrometers. The proton 90° pulse duration was typically between 8 and 10 μs. The sample temperature was regulated at +25 °C unless stated otherwise. Full experimental data and parameter sets can be found at DOI: 10.17632/p275tgwdv2.1.

### 3.1 Sample details

Four samples were used in this study: (1) 40 mM cyclosporin in benzene-*d*_6_; (2) 60 mM azithromycin in DMSO-*d*_6_; (3) 1% w/w *N*,*N*-diethylacetamide in DMSO-*d*_6_; and (4) 20 mM [Au_2_(μ-xantphos)_2_](NO_3_)_2_ in CD_2_Cl_2_. The latter complex was synthesised as reported earlier;^29^ all other compounds were obtained commercially and used as received.

### 3.2 NMR experiments

*T*_2_ measurements using CPMG, PROJECT and TRUE were performed on all four samples. CPMG and PROJECT experiments used delays *τ* of 200 and 800 μs respectively, with total relaxation-encoding times of 6.4, 12.8, 25.6, 51.2, 102.4, 204.8, 409.6, and 819.2 ms for samples (1) and (2); for (3) and (4) these values were adjusted as appropriate in the light of the relaxation rates for different temperatures. (In the cases of CPMG and PROJECT, the choice of relaxation-encoding time is constrained by the need to use an integer multiple of the basic echo time; there is no such limitation in the case of TRUE). The TRUE experiments on samples (1) and (2) used a 37 ms (50 Hz bandwidth) RSNOB pulse, which was sufficiently selective to suppress *J*-modulation for all signals in both samples used; for samples (3) and (4), 19.5 and 9.2 ms RSNOB pulses respectively were used. The coherence transfer pathway required in TRUE was enforced by 22 G cm^−1^, 1 ms field gradient pulses. The *T*_2_ experiment results were processed using monoexponential fitting of the amplitudes of selected resolved peaks, using Topspin 4.0.7 and VnmrJ 4.0 software for Bruker and Varian data respectively. Only well-resolved resonances were analysed, because overlap between the signals of different protons prevents reliable exponential fitting.

Variable-temperature 1D spectra were measured of samples (3) and (4); in the case of (3), time-shared homodecoupling was used to decouple the *N*,*N*-diethylacetamide methylene signals so that a two-site analysis of the signal bandshape could be performed. The irradiation of the methylene resonances used 200 Hz bandwidth DSNOB shaped pulses with Tycko 5 phase sequencing,^31^ at a 25% duty cycle. The DSNOB pulse shape was created using the standard VnmrJ pulse shaping software, with the appropriate offset and Bloch–Siegert shift compensation.

Selective inversion recovery (Hoffman–Forsén) experiments on samples (3) and (4) were carried out using 74 and 18.5 ms RSNOB pulses for (3) and (4), respectively, centred on each in turn of the methyl proton resonances. Eight and ten different recovery delays were used for (3) and (4) respectively; in each case the list of delays was tailored to the exchange rate at a given temperature. The data files at DOI: 10.17632/p275tgwdv2.1 include all the lists of delays used. Nonselective inversion recovery experiments on (3) and (4) used a 30 s relaxation delay and total recovery delays of 0.1, 0.25, 0.5, 0.75, 1, 2, 3, 5, 10, and 20 s. A single scan was used, with a 2 ms gradient pulse of 11 G cm^−1^ included in the recovery delay to avoid the need for phase cycling. A nonselective ^31^P inversion recovery experiment on (4) used broadband ^1^H decoupling with the WALTZ-16 sequence at 0.1 W power, corresponding to a 90° pulse duration of 145 μs. These unusual decoupling parameters were chosen to give sufficient decoupling of the small couplings to the aromatic protons without causing significant sample heating.

## 4 Results and discussion

The classic CPMG experiment (Fig. 1a) uses a train of repeated spin echoes. This results in a signal decay that is modulated to a greater or lesser extent by the effects of homonuclear couplings. If the interpulse delay 2*τ* is large compared to the inverse of the chemical shift difference 1/Δ*δ* between the coupled spins, full *J* modulation is seen. Conversely, if 2*τ* is small compared to 1/Δ*δ*, the transverse components of the coupling Hamiltonian are not averaged out, the spins become very strongly coupled, and *J* modulation is suppressed. The six significant problems with this approach – which nevertheless is commonly used – are (1) that contributions to relaxation from spin perturbations on a timescale long compared to 2*τ* are averaged out; (2) that refocusing rapidly compared to *J* suppresses any contribution to transverse relaxation from spin-lattice relaxation of coupled spins (the “scalar relaxation” described in Section 1 above); (3) that rapid refocusing causes isotropic mixing; (4) that it is often impractical to refocus sufficiently rapidly to suppress *J* modulation completely; (5) that the high radiofrequency power deposition can cause significant sample heating; and (6) that radiofrequency (*B*_1_) inhomogeneity distorts the amplitudes of the early echoes. Interestingly, *J* modulation can be quenched selectively for a given spin system at a much lower radiofrequency duty cycle, by careful optimisation of sequence parameters,^32,33^ allowing selective measurement of the transverse relaxation of a given proton.

The PROJECT experiment of Fig. 1b uses quadrature 90° pulses between echoes to exchange coherence between coupling partners. This refocuses the effects of homonuclear scalar coupling if 2*τ* ≪ 1/*J* (as opposed to the much more demanding requirement 2*τ* ≪ 1/Δ*δ* for the CPMG method), and hence requires much less frequent refocusing. This solves problems (4) and (5) with CPMG, and reduces problem (1), but problems (2) and (3) remain while problem (6) is more significant than for CPMG because far fewer echoes are needed. As with CPMG, in coupled spin systems scalar relaxation contributions are suppressed (problem 2) and the exchange of coherence between spins causes averaging (problem 3) of the transverse relaxation rates measured.

### 4.1 Simulation results

As explained in Section 1, spin–spin relaxation in coupled spin systems is complicated, in particular by the often-neglected contribution made to the transverse relaxation of a given spin by the longitudinal relaxation of any spins that are coupled to it.^14–16^ Just as the chemical exchange of spins between different environments broadens their NMR signals, so the exchange of magnetization between multiplet components caused by the change in spin state of a coupled spin broadens those lines. Because experiments using rapid refocusing suppress such effects (problem 2 above), such experiments tend to overestimate *T*_2_.

**Fig. 2:**
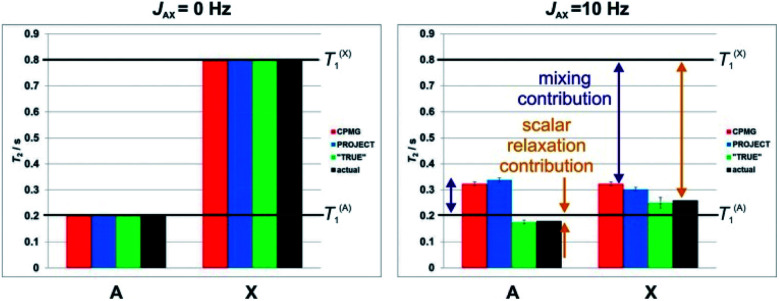
Simulated results of different pulse sequences for the measurement of transverse relaxation time, *T*_2_, in a two spin system (AX) with different spin-lattice relaxation times, *T*_1_. Apparent *T*_2_ values were obtained by exponential fitting of the results of numerical simulations of CPMG (red), PROJECT (blue), TRUE (green) experiments with *J*_AX_ = 0 Hz (left) and *J*_AX_ = 10 Hz (right), *T*_1_^(A)^ = 0.2 s, and *T*_1_^(X)^ = 0.8 s. The actual (black) values were determined from the linewidths in the simulated spectra. The contributions from scalar relaxation and mixing are highlighted by orange and purple double-headed arrows. Error bars show the uncertainty in *T*_2_ fitting that results from nonexponentiality of the decay. Note the unphysical implication of the CPMG and PROJECT data that the *T*_2_ of the more rapidly relaxing proton (A) is greater than its *T*_1_.

The origins of the overestimation of *T*_2_ by multiecho experiments such as CPMG and PROJECT are illustrated here by Spinach^28^ simulations of a simple system of two coupled spins-½ A and X. Random field relaxation in extreme narrowing, the simplest case, was assumed, with a coupling *J*_AX_ of either 0 (Fig. 2 left) or 10 Hz (Fig. 2 right), and different spin-lattice relaxation times *T*_1_ of 0.2 and 0.8 s. Using simulations allows very short pulse durations to be used, avoiding any effects due to finite pulse widths, and allows the actual *T*_2_ (*i.e.* the time constant for the loss of transverse magnetisation during free precession) to be deduced directly from the peak width. This provides a benchmark for evaluating the results of CPMG, PROJECT and TRUE.

Fig. 2 shows the apparent *T*_2_ values obtained using CPMG, PROJECT and TRUE simulations. All three methods gave the same, correct, result (*T*_2_ = *T*_1_) if the coupling *J*_AX_ was zero. For nonzero *J*, however, only TRUE gave the correct value (1/*T*_2_^A^ = 1/*T*_1_^A^ + 1/(2*T*_1_^X^), since the intrinsic *T*_2_ is equal to *T*_1_ here). The scalar contribution here is the second term; where the two coupled spins have different *T*_1_ values, it is greater for the spin (here X) which has the greater *T*_1_. In Fig. 2, the scalar relaxation contributions are marked by orange double-headed arrows. The CPMG and PROJECT simulations gave results that consistently overestimated *T*_2_, partly because of the suppression of the scalar relaxation contributions, and partly because of the exchange of coherence between coupled spins (problem 3, the mixing contribution). Problems (2) and (3) can have opposite effects, as seen for the more rapidly relaxing spin A. Despite CPMG and PROJECT both suppressing the scalar relaxation contribution, they report slightly different apparent *T*_2_s with non-zero *J*_AX_ coupling, because of the difference in mixing mechanism noted in Section 1.

Where, as here, two coupled spins have very different values of *T*_1_, the averaging of relaxation rates between coupled spins caused by the mixing can lead both CPMG and PROJECT to show an apparent *T*_2_ for the more rapidly relaxing spin (here A) which is actually greater than *T*_1_. This is impossible in the relaxation regime used in these simulations (and occurs only under very exotic conditions in practice), and highlights the tendency of these experiments to give misleading results. Taken together, these problems mean that in complex coupled spin systems the results of CPMG and PROJECT experiments can be grossly unreliable, and their interpretation very problematic, particularly where there is a range of *T*_1_ values.

### 4.2 Experimental comparison of relaxation experiments

**Fig. 3:**
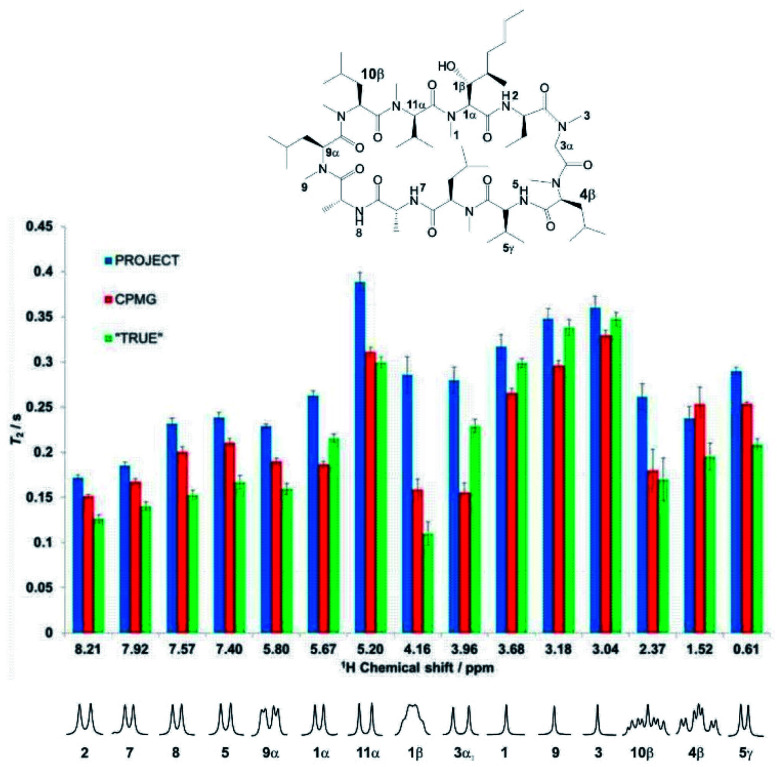
Relaxation data (*T*_2_) for selected well-resolved protons of cyclosporin (1) in benzene-*d*_6_. Data shown as blue, red, and green bars are the results of PROJECT, CPMG, and TRUE *T*_2_ experiments respectively. The error bars indicate the error estimated in the fitting, but do not take into account the systematic errors due to mixing and scalar relaxation in CPMG and PROJECT experiments. The outliers in the CPMG experiment are due to residual *J* modulation, which makes the monoexponential fitting unreliable. Multiple sources of systematic errors may cancel accidentally (as *e.g.* for proton 10β).

Experimental results reinforce the conclusions above and illustrate the problems with multiple refocusing methods such as CPMG and PROJECT. Fig. 3 compares the measured values of *T*_2_ obtained for the cyclosporin sample, (1), using the CPMG, PROJECT and TRUE methods. The atom labelling in Fig. 3 follows that used in the full assignments reported earlier.^34^ Expanded individual proton multiplets are plotted below the histogram to aid discussion.

Substantial differences are seen, in many cases much larger than the estimated uncertainties, between the different values of *T*_2_ obtained for a given proton. As expected, the PROJECT experiment, which efficiently suppresses both *J* modulation and the effects of scalar relaxation, shows the highest apparent *T*_2_ values throughout. For the most part the TRUE experiment returns the lowest values, again as expected given that the other two methods suppress the scalar relaxation. CPMG generally shows values intermediate between PROJECT and TRUE, but in the case of proton 3α (3.96 ppm) it gives the lowest value of the three methods. Examination of the experimental data shows that this is due to residual *J* modulation, despite the very low delay *τ* used of 200 μs, causing rapid signal decay. In principle this modulation could be reduced by decreasing the echo time still further, but in practice the radiofrequency power deposition is already high and any more would be excessive. The effect of radiofrequency heating can be seen in the change in chemical shift observed for the amide proton, and is also likely to be the cause of the minor but systematic difference between the *T*_2_ values reported by CPMG for the uncoupled methyl protons (H-1, H-9 and H-3) and those found with PROJECT and TRUE. The extent of residual *J* modulation depends on the chemical shift difference between the coupled spins and on the magnitude of the coupling. The apparent agreement between CPMG and TRUE for proton 10β (2.37 ppm), which like 3α shows a large *J*, is alas illusory: again, residual *J* modulation is contributing substantially to the measured signal decay.

Incomplete suppression of *J* modulation is also likely to be the main source of the systematic differences between CPMG and PROJECT results for the four amide proton doublets (2, 5, 7, 8). The effects of mixing and suppression of scalar relaxation contributions are difficult to separate unambiguously here, but the former effect is likely to be the main contributor to the overestimation of *T*_2_ by PROJECT compared to the actual rate of decoherence measured by TRUE. Since the relaxation rates of the amide protons are greater than those of their coupling partners (CH_α_s), we expect scalar contributions to be less important (note the significant difference observed between A and X in the AX spin system simulation of Fig. 2), but mixing with the more slowly relaxing alpha protons will increase the apparent amide *T*_2_s in PROJECT.

A similar pattern is seen for CPMG, PROJECT and TRUE measurements on sample (2), azithromycin in DMSO-*d*_6_; results are summarised in Fig. S2 of the ESI,† using the resonance assignments previously reported.^35^ The only protons for which CPMG, PROJECT and TRUE result in the same *T*_2_ within experimental error are those of the H-2′ hydroxyl group. These have no resolvable couplings and hence show neither scalar relaxation nor mixing effects. As with cyclosporin, there are a number of sites (*e.g.* H-1′, H-3, H-5′, H-2′, H-4′′), where the PROJECT and CPMG *T*_2_s differ significantly because of residual *J* modulation in the CPMG experiment. The suppression of scalar contributions (*e.g.* for H-2′ and H-5′) is evident from the systematic overestimation of *T*_2_s in PROJECT.

**Fig. 4:**
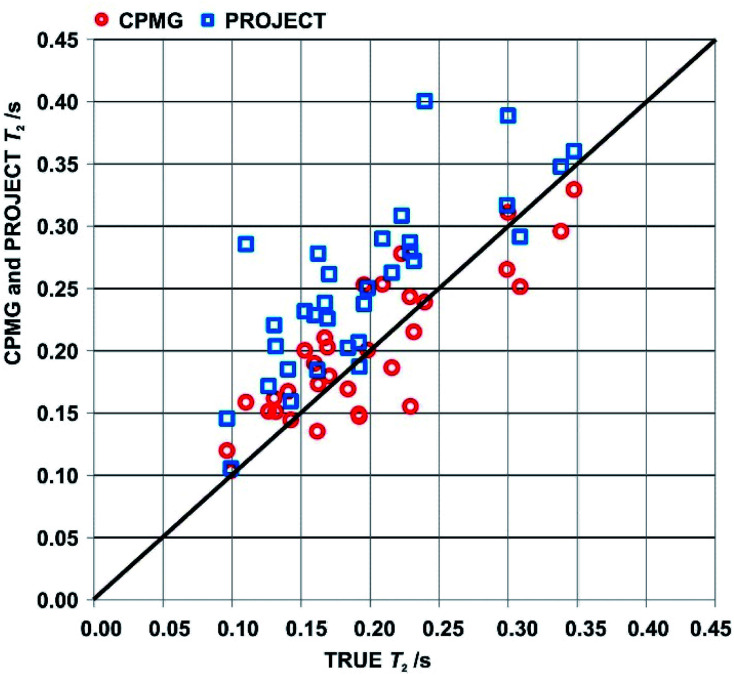
Scatter plot of apparent *T*_2_s measured using CPMG (red circles) and PROJECT (blue rectangles) with respect to the TRUE *T*_2_s. Data are for resolved proton signals in the spectra of samples (1) and (2), cyclosporin in benzene-*d*_6_ and azithromycin in DMSO-*d*_6_.

An overview of the comparison between CPMG, PROJECT and TRUE results for the two samples is provided by the scatter plot in Fig. 4 of apparent *T*_2_s from CPMG and PROJECT with respect to the TRUE *T*_2_s. As noted in Section 3, only well-resolved resonances were studied. Significantly more resonances could be resolved if pure shift versions of the three experiments were used; in the case of TRUE the sensitivity penalty would be much smaller than for CPMG and PROJECT because it has already been partly paid by the use of the ZS sequence element. In all three cases the sensitivity penalty could be reduced by the use of real-time^36,37^ or semi-real-time^38^ acquisition methods.

The scattergram allows a number of general conclusions to be drawn. First, PROJECT tends to show longer apparent transverse relaxation times than TRUE, *i.e.* the majority of the data points are above the diagonal line. Second, CPMG tends to show shorter apparent transverse relaxation times than PROJECT. The first observation is a consequence of the suppression of scalar relaxation, exchange and slow motion contributions to transverse relaxation in PROJECT. The same is true of CPMG, but the second observation arises because many of the coupled spin systems in cyclosporin span too wide a range of chemical shifts for *J* modulation to be fully suppressed with CPMG even at the short (*τ* = 200 μs) echo time used, leading to extra signal decay in CPMG. The PROJECT results, in contrast, show essentially perfect suppression of *J* modulation, as expected given its much less demanding requirement for the echo time 2τ (2*τ* ≪ 1/*J* as opposed to 2*τ* ≪ 1/Δ*δ*). The better agreement of CPMG with TRUE is thus an artefact, a consequence of the limited radiofrequency duty cycle usable in CPMG.

### 4.3 Amide rotation in *N*,*N*-diethylacetamide

Accurate measurements of transverse relaxation can be useful for determining the rates of exchange processes, and hence the activation energies of the underlying processes. Here we describe a new method to estimate exchange rates from relaxation data below coalescence temperature, using the classic example of hindered amide rotation as proof of principle. The reliability of activation energy measurements is often limited by the range of temperatures for which experimental data can be obtained. The complementary use of different experimental methods extends this temperature range, and greatly improves accuracy. Amide rotation has been extensively studied with a range of methods, including bandshape analysis^39^ for processes that are relatively fast compared to transverse relaxation, and selective inversion recovery (also known as the Hoffman–Forsén experiment, and in 2D form as EXSY) for processes slow compared to transverse relaxation.^40,41^ Here we show that the TRUE method offers a direct route to rate constants in the latter range, without the need for special data fitting, and, importantly, without the constraint that the multiplets of interest be well resolved.

Transverse relaxation of the A proton in an exchanging AX spin system in extreme narrowing with *J T*_1_^X^ ≫ 1 is well represented by exponential decay with a rate constant 1/*T*_1_^A^ + 1/(2*T*_1_^X^) + *k*, where *k* is the rate constant for the exchange. Therefore, by combining the results of TRUE and inversion recovery experiments the rate constant for exchange can be separated from the intrinsic transverse relaxation. In more complex spin systems the correction for scalar relaxation needs to include the effects of all passive spins. If the assumption of extreme narrowing is not valid, the 1/*T*_1_^A^ term (but not the 1/(2*T*_1_^X^), which remains valid) can be estimated from the apparent *T*_2_ measured by CPMG, since this suppresses both the exchange and the scalar contribution. Where the condition *J T*_1_^X^ ≫ 1 is not met it is still possible, though less straightforward, to disentangle exchange from the other contributions to *T*_2_, provided the relevant coupling constants are known.

**Fig. 5:**
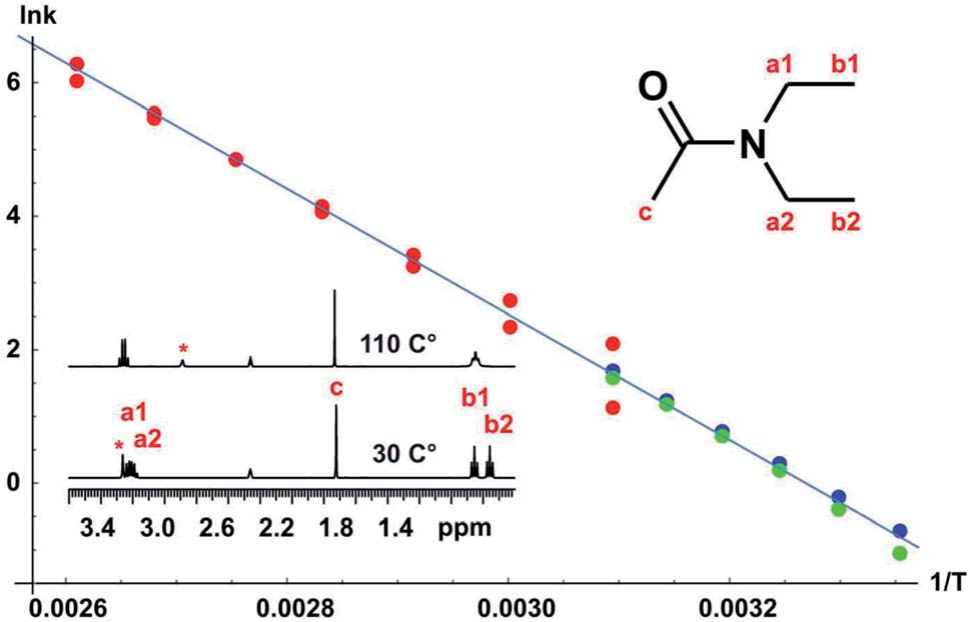
Arrhenius plot for *N*,*N*-diethylacetamide. ^1^H NMR spectra corresponding to slow and fast exchange regimes are shown. The temperature dependence of the exchange rate was estimated using bandshape analysis (red), Hoffman–Forsén experiments (green), and TRUE experiments (blue), all for the methyl protons. Bandshape analysis results show exchange rates found using upper and lower limits of the experimental linewidth imposed by field inhomogeneity, transverse relaxation and imperfect decoupling. The activation energy *E*_a_ obtained was 78.3 ± 1.4 kJ mol^−1^.

A full set of experiments was carried out for the classic case of *N*,*N*-diethylacetamide, using bandshape analysis, the Hoffman–Forsén experiment, and TRUE, the latter showing good consistency with the Hoffman–Forsén results. In this system the multiplets for the methylene protons (a1 and a2) overlap, preventing the use of Hoffman–Forsén or EXSY experiments, but the TRUE measurements are still straightforward because individual multiplet components are resolved. The methyl signals are well resolved, and inter-methyl cross-relaxation is negligible, so the Hoffman–Forsén experiment provides a direct comparison with TRUE. The resulting Arrhenius plot showing data from all three methods is shown in Fig. 5, and gives an activation energy for the amide rotation of 78.3 ± 1.4 kJ mol^−1^. Full details and results are given in the section 3 of the ESI.†

Bandshape fitting was carried out in Mathematica, using direct analytical solutions of the Bloch–McConnell equations, for the methyl proton resonances. A simplification that is often used is to fit exchange-broadened multiplets as if they were singlets, but this leads to overestimation of exchange rates. Here, time-shared homodecoupled acquisition was used to collapse the triplets of the methyl signals to singlets, so that a two-site exchange model is directly applicable. Homonuclear decoupling is not perfect, so the residual linewidth contributed needs to be taken into account, as does the contribution from field inhomogeneity. This is in addition to the effect of transverse relaxation, which in extreme narrowing is known from *T*_1_. The inclusion of such contributions to the linewidths and bandshapes actually measured significantly improves the reliability of analysis at lower temperatures, in the slower exchange regime, but is often neglected in applications of bandshape analysis. It is not possible to measure all these contributions directly, so fitting was performed twice, for upper and lower estimates of the instrumental linewidth. This is much more informative than the statistics of the fitting process; because these assume a normal distribution, they lead to error estimates for *k* that are far smaller than the systematic uncertainties introduced by the experimental linewidth.

Fitting of the results of Hoffman–Forsén experiments was again carried out in Mathematica using direct analytical solutions of the Bloch–McConnell equations (see DOI: 10.17632/p275tgwdv2.1). Simultaneously fitting the results for inversion of each exchanging signal in turn improves the accuracy and reliability of fitting. The main limitation of the Hoffman–Forsén method is the need to invert the exchanging resonances selectively. Here this is straightforward for the well-resolved methyl signals, but is not possible for the methylene quartets, which are interpenetrated.

An alternative to the Hoffman–Forsén method for determining rate constants below coalescence temperature is to measure transverse relaxation rates. For this purpose it is crucial to measure the actual rate of decoherence, for example with the TRUE experiment, without the bias caused by the mixing of coherences of coupled spins and the suppression of slow relaxation contributions, but it is important to include the effects of scalar relaxation in the analysis. The use of conventional experiments such as CPMG and PROJECT can cause significant systematic errors here. A great advantage of *T*_2_ measurement is its applicability to complex spectra such as the interpenetrating methylene quartets in this example. As discussed in Section 1, the true transverse relaxation rate of a coupled proton contains scalar relaxation contributions from all the protons with which it has a resolved coupling. These contributions can be found by measuring *T*_1_ using conventional broadband inversion recovery experiments. For the methyl and methylene protons in *N*,*N*-diethylacetamide, exchange rates can therefore be found by taking the measured transverse relaxation rate and subtracting both the intrinsic relaxation contribution 1/*T*_2_^0^ (= 1/*T*_1_ in extreme narrowing, as here) and the scalar relaxation contribution 1/*T*_1_^methylene^ and 3/(2 *T*_1_^methyl^) respectively, as described in the section 3 of the ESI.† As Fig. 5 shows, there is good agreement between Hoffman–Forsén and TRUE measurements of exchange rate using the methyl protons of *N*,*N*-diethylacetamide.

There are two important limitations on the use of transverse relaxation measurements to determine exchange rates of coupled protons. The first is that all the scalar relaxation contributions need to be quantifiable, and the second is that they need to be small compared to the coupling constants *J* so that modulation of the decay curve remains small. The agreement between Hoffman–Forsén and TRUE measurements is less good in the case of the methylene protons of *N*,*N*-diethylacetamide, where there is a small but statistically significant extra relaxation contribution of about 0.7 s^−1^ that is not accounted for (see ESI†). There will here be a scalar contribution from the adjacent ^14^N nucleus. This is “scalar relaxation of the second kind”, as the spin-lattice relaxation of the ^14^N is fast compared to the coupling constant, but the effect should be quite small as the ^14^N spectrum shows a linewidth at half height of *ca*. 800 Hz, corresponding to a ^14^N *T*_1_ of about 400 μs, and the two-bond coupling *J*_NH_ will be small. The remaining contribution is probably from the four-bond coupling between *N*-methyl protons across nitrogen.

### 4.4 Conformational interconversion in [Au_2_(μ-xantphos)_2_](NO_3_)_2_

As a more challenging example we include a study of the complex [Au_2_(μ-xantphos)_2_](NO_3_)_2_, which adopts a helical folded structure, in dichloromethane solution. Interconversion between mirror-image conformers causes temperature-dependent line broadening; the activation energy barrier for this process has previously been investigated using bandshape analysis.^29^ The full assignment in the slow exchange regime, and conformational analysis with NOE and experimental proof for the aurophilic interaction, *i.e.* a bond between the two gold nuclei, were also reported.^30^ The activation energy was estimated using bandshape analysis for the methyl proton signals and the ^31^P spectra. Here we provide a more comprehensive analysis of the exchange process by using a combination of bandshape analysis, Hoffman–Forsén experiments and TRUE experiments.

**Fig. 6:**
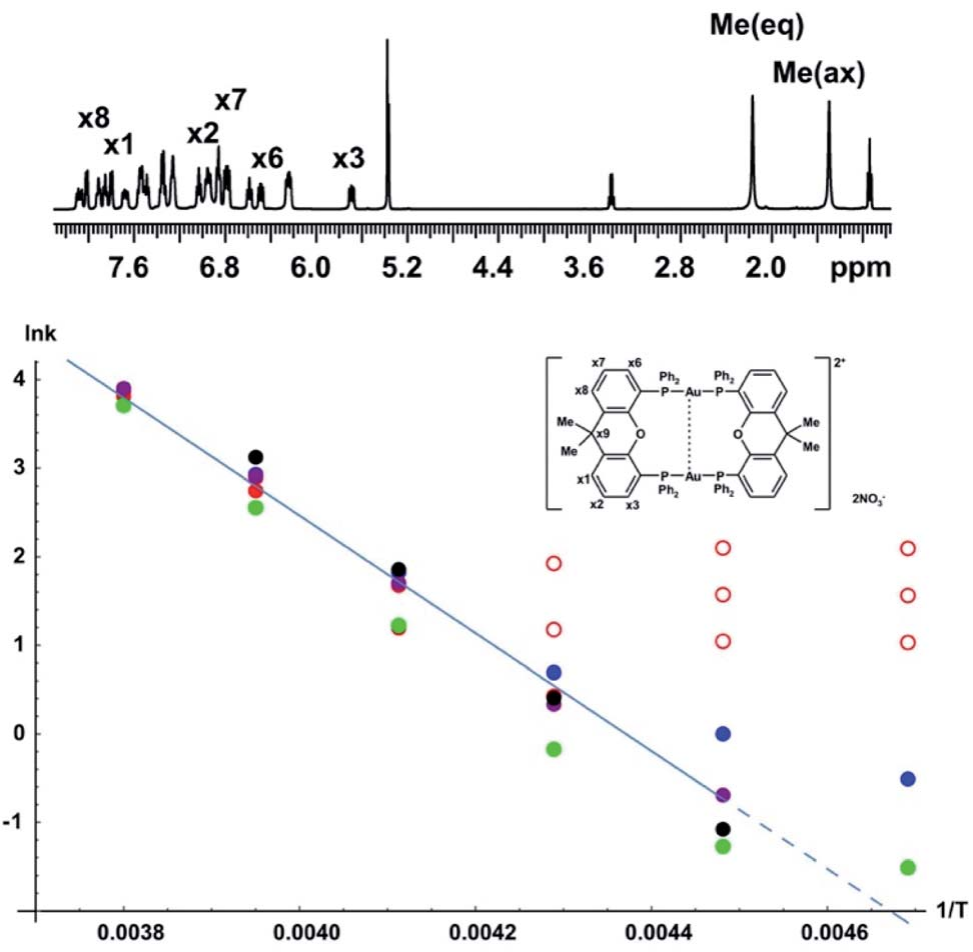
Arrhenius plot for conformational interconversion of [Au_2_(μ-xantphos)_2_](NO_3_)_2_ in CD_2_Cl_2_. The assigned structure and ^1^H NMR spectrum in slow exchange at −60 °C are shown as insets; the full assignment has been published elsewhere.^30^ The temperature dependence of the exchange rate was determined using lineshape analysis (red), Hoffman–Forsén experiments (green), and TRUE experiments on equatorial methyl, axial methyl and *x*_3_ aromatic resonances (blue, purple and black respectively). Data points used for least squares fitting to the Arrhenius law are shown with filled symbols, those omitted from fitting with open symbols; the activation energy *E*_a_ obtained was 55 ± 5 kJ mol^−1^.

The schematic structure, and the assignments of the protons relevant for this study in the low temperature spectrum, are shown as insets in Fig. 6. The complex adopts a helical conformation with *C*_2_ symmetry, which is identical to the solid-state structure determined by single crystal X-ray diffraction data.^29^ This causes the four phenyl rings to be non-equivalent, and the ^31^P spectrum shows an AA′BB′ spin system with a large two-bond coupling and small ^3^*J* couplings mediated by the aurophilic interaction. With increasing temperature, the two enantiomers interconvert, exchanging the following pairs of non-equivalent groups: (1) phosphoruses A and B, (2) the equatorial and axial methyl groups in the xanthene ligand, (3) the phosphine phenyl groups, (4) and the two pairs of xanthene phenyl rings. The *ortho* and *meta* protons of the phosphine phenyl rings also show restricted rotation. The activation energy for the main conformational exchange of the helical enantiomers can be determined by investigating the temperature dependence of the exchange rate measured for a suitable pair of nuclei such as the methyl protons, or the aromatic proton pairs in the xanthene ligand. In the current study the available temperature range did not allow study of the restricted rotation of the phenyl rings.

The Arrhenius plot of Fig. 6 was constructed by combining data from bandshape analysis, selective inversion recovery, and TRUE *T*_2_ relaxation measurements for both the methyl groups and the *ortho* protons (*x*_3_/*x*_6_) in the xanthene ligand. Full results for all three experiments are again given in the section 4 of the ESI.† The exchange rates estimated from bandshape analysis of the methyl groups at lower temperatures deviated significantly from those found for the other two groups. This is because the linewidths of the methyl groups include a significant extraneous broadening which is not accounted for in the bandshape analysis. At first sight bandshape analysis of the exchanging methyl proton resonances should be similar to the classic example of amide rotation. However, even after taking into account transverse relaxation and instrumental broadening bandshape fitting did not provide reliable results below −30 °C, as seen in Fig. 6, and results below this temperature were omitted from the Arrhenius fitting.

The problems here are caused by unresolved long-range proton–proton couplings. A long-range optimised gradient-selected COSY experiment (see ESI Fig. S12†) at −50 °C confirmed the presence of both methyl–methyl and methyl–phenyl (*x*_1_ and *x*_8_) couplings, the latter causing a slight differential broadening of the two methyl peaks. (Interestingly, a similar differential broadening can be seen in the classic example of amide rotation, dimethylacetamide. In that case the long-range coupling between the *N*-methyls and the carbonyl methyl is very different for the two exchanging methyls, leading to significantly different linewidths, but this appears to have escaped comment in previous studies.)

Selective inversion recovery and relaxation measurements are generally much more reliable in slow exchange than bandshape analysis. The use of TRUE here, as well as extending downwards the temperature range over which reliable data can be obtained, allows complex multiplet signals, such as those of the aromatic protons here, to be used for analysis. The Hoffman–Forsén experiment gives good results for the methyl signals here, as these are well-resolved. The application of TRUE requires a slightly different approach to that used for *N*,*N*-diethylacetamide, as this sample is out of extreme narrowing, as evidenced by the increase in *T*_1_ with decreasing temperature (ESI Tables S8 and S9†). Here a CPMG experiment was used to estimate the intrinsic transverse relaxation rate 1/*T*_2_^0^ instead of deducing this from *T*_1_. Scalar relaxation contributions were calculated as previously, using the measured *T*_1_ values for the passive spins. The long-range couplings to and between the methyl protons are unresolved, so the contribution from scalar relaxation is small. In the case of aromatic proton *x*_3_, the scalar contribution includes two significant terms, one for the phosphorus coupling and one for the proton coupling partner *x*_2_, the coupling to *x*_1_ being negligible. In summary, the experiments required are thus CPMG and TRUE experiments for the exchanging proton resonance, and inversion recovery for all of its resolved coupling partners, including any heteronuclei. (A familiar consequence of scalar relaxation caused by heteronuclei is the broad multiplet structure seen for the residual proton signals of deuteriated solvents such as DMSO-*d*_6_ and CD_2_Cl_2_ here). At −60 °C the Hoffman–Forsén data deviate substantially from the linear trend at higher temperature, because of the presence of through-space magnetization exchange by cross-relaxation. This is consistent with the previously observed strong, negative NOEs between the methyl groups in the slow exchange limit (below −80°).^30^ The TRUE results are not affected by this, but have a high uncertainty as the intrinsic *T*_2_ relaxation is fast compared to exchange. The impact of unresolved couplings on exchange rates derived from TRUE is smaller than that on bandshape analysis; this is the main reason for the significant differences between them at lower temperature. All data at −60 °C were therefore omitted from the Arrhenius fit, in addition to the bandshape results for −40 and −50 °C. This example demonstrates the robustness of the TRUE method compared with the complementary methods of bandshape analysis and Hoffman–Forsén (selective inversion) experiments, which are biased by unresolved couplings and by cross-relaxation, respectively.

## 5 Conclusions

In summary, CPMG and PROJECT methods are widely used for measurement of apparent spin–spin relaxation times *T*_2_ (and for other purposes such as *T*_2_ weighting), but in coupled spin systems they both average the relaxation of coupled spins, and frequently overestimate *T*_2_. In addition, CPMG often struggles to suppress *J* modulation, particularly at high field where peak separations are large. In contrast the new method presented here, Transverse Relaxation Unmodulated Echo (TRUE), measures the true decoherence rate, uncontaminated by the effects of repeated refocusing. Relaxation measurements using such methods have the potential to expand greatly the range of species for which NMR can be used to determine the rates and activation barriers of chemical processes, by enabling more reliable measurements on coupled spins. This is particularly valuable for the determination of activation barriers by NMR, where it is important to be able to make measurements over a wide temperature range. The results of the TRUE method can also be used to correct for the effects of transverse relaxation on signal intensities in a wide range of multiple pulse NMR experiments, both 1D and multidimensional, improving the quality of quantification obtainable with such methods.

## Data availability

Further experimental results, pulse sequence code for Bruker and for Varian/Agilent spectrometers, and Matlab SPINACH simulation code are given in the ESI.† All raw experimental data, pulse sequence code and software can be downloaded from DOI: 10.17632/p275tgwdv2.1.

## Author contributions

Conceptualization: GAM, PK, MN, LC; formal analysis, GAM; funding acquisition: GAM, MN, LC; investigation: PK, GDP, AD; methodology: PK, GAM; project administration: GAM, MN, LC; resources: AD, PK; software: GAM, PK; supervision: GAM, MN, LC; writing – original draft: PK, GAM. All authors proofread, commented on, and approved the final version of the manuscript.

## Conflicts of interest

There are no conflicts to declare.

## Supplementary Material

SC-012-D1SC03391C-s001
