# Supplementary material for: Broadband measurement of true transverse relaxation rates in systems with coupled protons: application to the study of conformational exchange
Source: Chem Sci. 2021 Aug 3;12(34):11538–47. doi: 10.1039/d1sc03391c (PMC8447259; doi:10.1039/d1sc03391c)
Supplement: SC-012-D1SC03391C-s001 [file SC-012-D1SC03391C-s001.pdf]

Electronic Supplementary Material (ESI) for Chemical Science.
This journal is © The Royal Society of Chemistry 2021

Electronic Supplementary Information

Broadband Measurement of True Transverse Relaxation Rates in Systems with Coupled Protons: application to the study of conformational exchange

Peter Kiraly[a†], Guilherme Dal Poggetto[a‡], Laura Castañar[a], Mathias Nilsson[a], Andrea Deák[b] and Gareth A. Morris*[,a]

[a] School of Chemistry, University of Manchester, Oxford Road, Manchester M13 9PL, UK. E-mail: g.a.morris@manchester.ac.uk

[b] Research Centre for Natural Sciences, Institute of Materials and Environmental Chemistry, "Lendület" Supramolecular Chemistry Research Group, 1117 Budapest, Magyar Tudósok körútja 2, Hungary

[†] Present address: JEOL UK Ltd., Bankside, Long Hanborough, OX29 8SP, UK

[‡] Present address: Institute of Chemistry, University of Campinas (UNICAMP), PO BOX 6154, Campinas (SP), CEP 13083-970, Brazil

Full experimental data and analysis software can be downloaded from DOI: 10.17632/p275tgwdv2.1.

Table of Contents

1. Scalar relaxation effects in the intermediate exchange regime 3

2. Relaxation data for azithromycin 5

3. Study of amide rotation in *N*,*N*-diethylacetamide 6

4. Study of conformational exchange in $[Au_2(\mu\text{-xantphos})_2](NO_3)_2$ complex..................................14

5. Pulse programs for Bruker spectrometers..20

6. Pulse programs for Varian/Agilent spectrometers 22

7. Matlab source code for numerical simulations with the spinach program libraries 25

1. Scalar relaxation effects in the intermediate exchange regime

If the inverse $1/T_1^X$ of the X spin-lattice relaxation time is not small compared to $2\pi J_{AX}$, a small modulation is superimposed on the exponential relaxation of transverse magnetization. This is because X spin transitions only cause decoherence of A spin coherence that is antiphase with respect to spin X, since when the A spin doublet components are in phase exchanging them has no effect. The rate of loss of coherence in the AX example therefore oscillates between $1/T_1^A$ and $1/T_1^A + 1/T_1^X$, with period J_{AX}, as the doublet components go in and out of phase. The oscillation between these can be observed directly both in spin-selective spin echo experiments and in the TRUE experiment, which is a broadband analogue of the selective spin echo. It is important to distinguish between the small oscillations caused by scalar relaxation and the much larger oscillations, in which signals can change sign, caused by classical J modulation, as for example in the simple (Carr-Purcell Method A) spin echo. The net effect of scalar relaxation is an equal mixture of "in-phase relaxation" and "antiphase relaxation". For small modulation index ($1/T_1^X < J_{AX}$) the average magnetization decay is well approximated by an exponential with decay constant $1/T_1^A + 1/(2T_1^X)$, but in extreme cases significant deviations from exponential decay can be seen.

One consequence of scalar relaxation is that real multiplets in experimental spectra are not simply composed of absorption mode Lorentzian lines, as is almost universally assumed (including in standard spectral simulation software). As is (moderately) well known, the bandshape of two chemically exchanging resonances in intermediate exchange (i.e. around coalescence) is not just the sum of two absorption mode Lorentzians, but rather of two Lorentzians with equal and opposite phase deviations from pure absorption mode. It is the combined effect of the two phase shifts that gives rise to the familiar appearance in which the signal amplitude between the maxima is higher, and that outside the peaks lower, than is expected for two overlapping Lorentzian peaks. Exactly the same thing happens with scalar relaxation:

the doublet of spin A in an AX spin system shows the same type of bandshape as in chemical exchange, as the magnitude of the scalar relaxation contribution made by spin-lattice relaxation of spin X to the spin-spin relaxation of spin A approaches J_{AX}.

Figure S1 shows the result of a Spinach simulation of the A doublet of an AX spin system with J_{AX} = 5 Hz, T_1^A = 1 s and T_1^X = 0.1 s, with the calculated spectrum fitted with two absorption mode Lorentzians (a,b) and with two Lorentzians with equal and opposite phase shifts (c,d). As can be seen, the residuals show significant errors in the former case but a perfect fit in the latter. In this example the parameters were chosen to give a particularly strong effect, but particularly in high dynamic range spectra the lineshape changes caused by scalar relaxation are a potential limiting factor in global spectral fitting software. The impact of homonuclear scalar relaxation on multiplet bandshapes is almost always neglected, but its effect has been noted in macromolecular structure determination (G. S. Harbison, *J. Am. Chem. Soc.*, 1993, 115, 3026-3027). It is much more commonly recognised in the heteronuclear case, especially where quadrupolar relaxation leads to short T_1s (see e.g. R.K. Harris, *Nuclear Magnetic Resonance Spectroscopy*, Pitman, London, 1983).

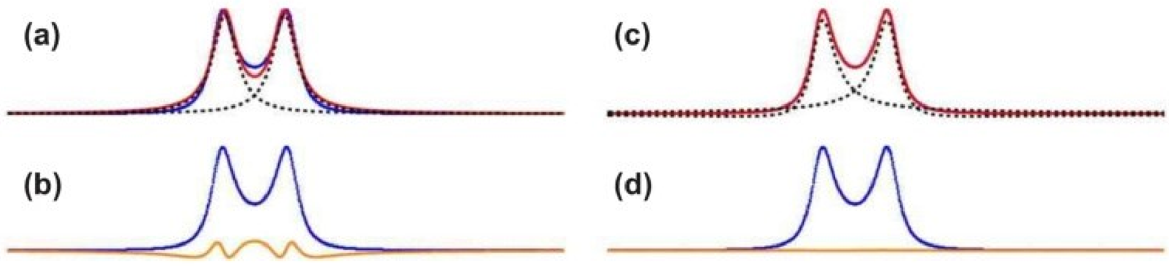

Fig. S1 Doublet of spin A from a conventional 1D ^{1}H NMR spectrum simulated using Spinach for an AX spin system with J_{AX} = 5 Hz, $\nu_A - \nu_X$ = 500 Hz, T_1^A = 1.0 s, T_1^X = 0.1 s. In (a) and (c) the simulated spectrum, fitted curve and individual peaks are shown in blue, red and dashed grey respectively; in (b) and (d), the simulated spectrum and residuals are shown in blue and orange respectively. Fitting used the sum of two pure absorption mode Lorentzian lines of equal amplitudes in (a) and (b); in (c) and (d) equal and opposite phase shifts of the two Lorentzian lines were allowed.

2. Relaxation data for azithromycin

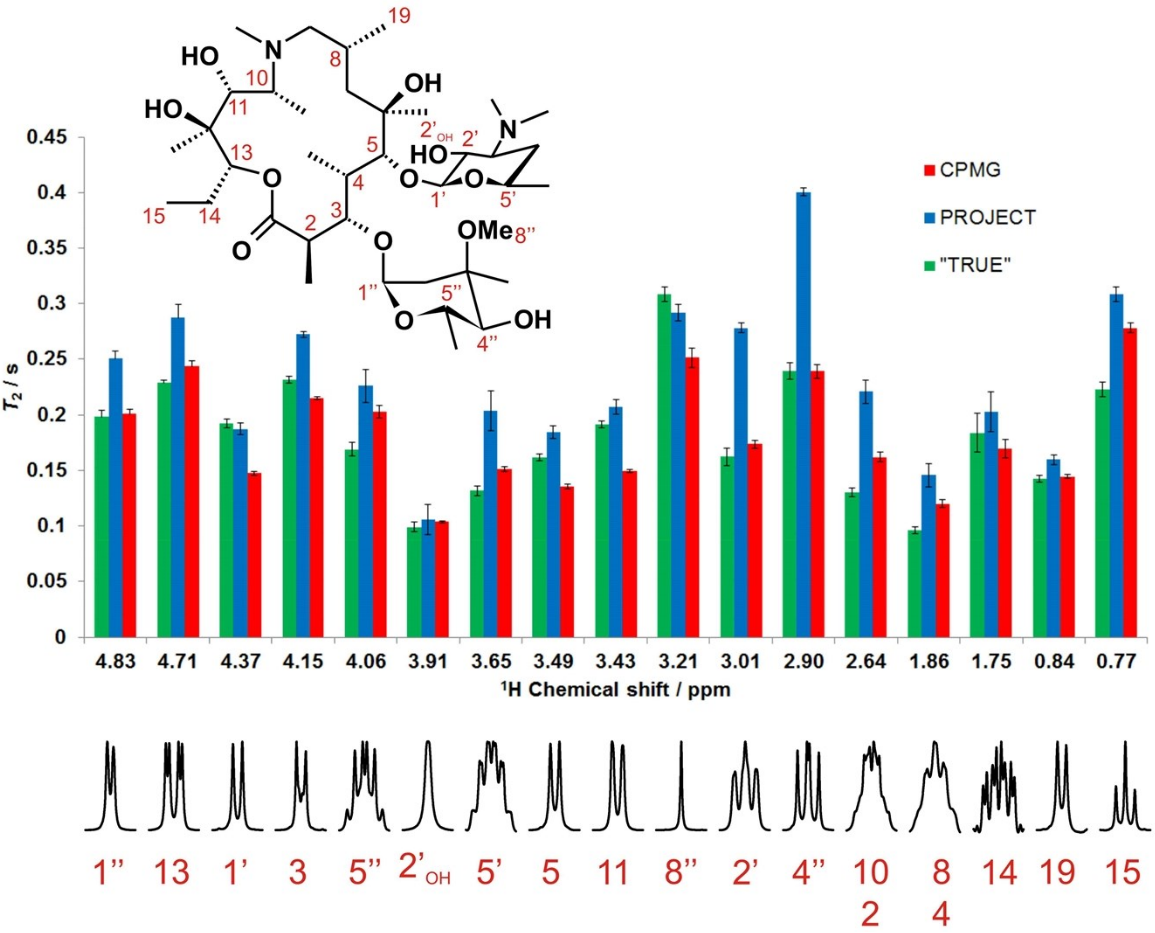

Fig. S2 Relaxation data (apparent T_2) for selected well-resolved protons of azithromycin in DMSO-d_6. Data shown as red, blue and green bars are the results of CPMG, PROJECT, and TRUE T_2 experiments respectively. The error bars indicate the errors estimated in exponential fitting, and assume a normal error distribution. The outliers in the CPMG data are the result of residual *J* modulation, which makes exponential fitting unreliable for these sites.

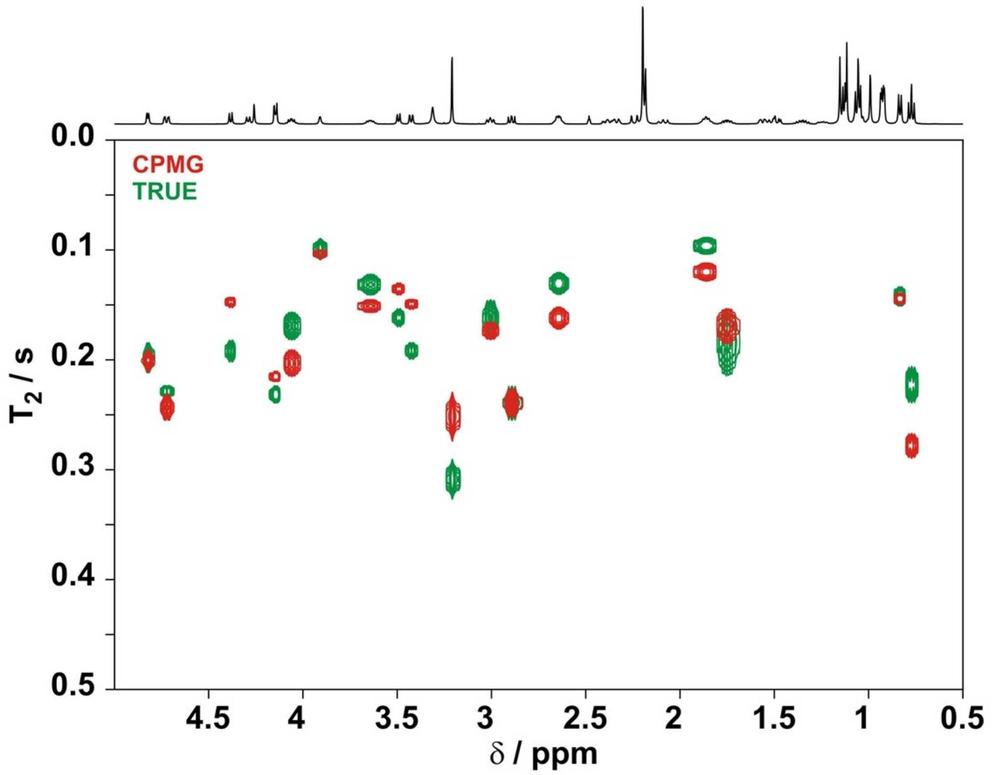

Fig. S3 ROSY (Relaxation Order Spectroscopy) plot for azithromycin in DMSO-d_6, comparing the results of CPMG (red) and TRUE (green) T_2 experiments.

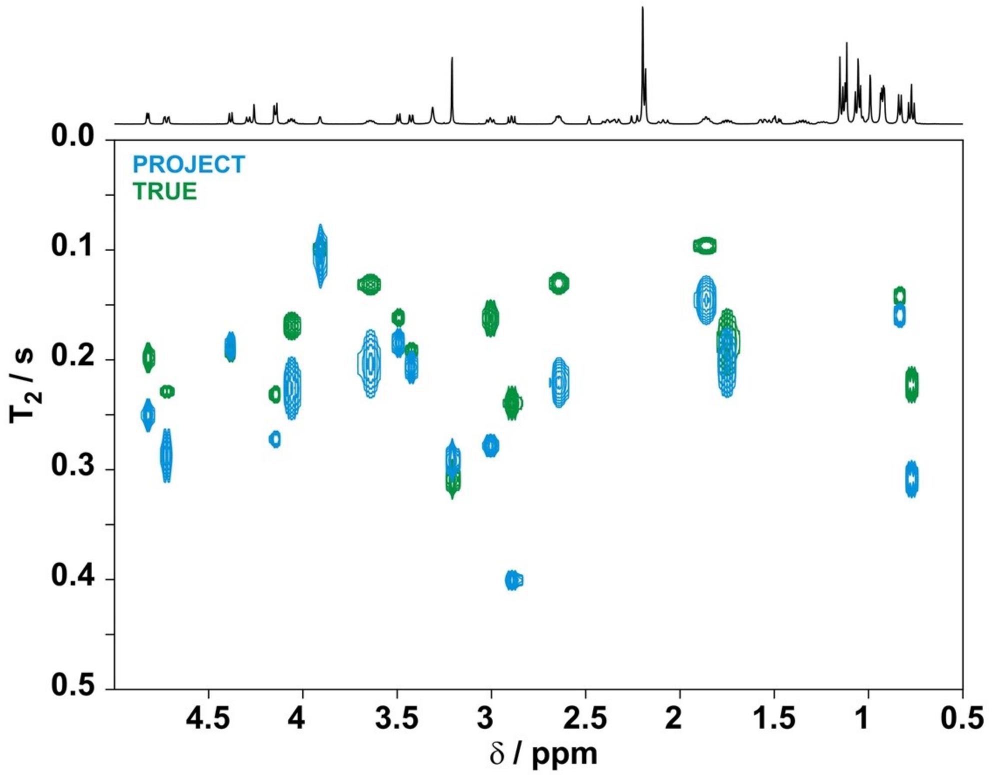

Fig. S4 ROSY (Relaxation Order Spectroscopy) plot for azithromycin in DMSO-d_6, comparing the results of PROJECT (blue) and TRUE (green) T_2 experiments.

3. Study of amide rotation in *N,N*-diethylacetamide

To test the consistency between kinetic data obtained with TRUE experiments and with conventional methods, a full analysis of the kinetics of exchange between the methyl resonances of *N*,*N*-diethylacetamide (DEA) was performed using the new TRUE method, bandshape analysis, and selective inversion recovery (the Hoffman-Forsén experiment). Measurements with the latter two techniques used time-shared homonuclear decoupling of the methyl from the methylene resonances in order to avoid the complications caused by multiplet structure; no decoupling was used in TRUE.

400 MHz 1D ^{1}H spectra of DEA in DMSO-d_6 at nominal temperatures from 50 to 110 °C were recorded on a Varian INOVA 400 spectrometer, using homonuclear time-shared decoupling irradiation centred midway between the two overlapping methylene resonances. Bandshape analysis in Mathematica used analytical solutions of the Bloch equations for two-site exchange assuming equal transverse relaxation rates for the two exchanging methyl resonances. The small temperature dependence of chemical shift difference (ca. –0.2 Hz/°) was determined from the spectra for temperatures in the range 50 – 80 °C, and included in the analysis. The dominant source of uncertainty in the experimental data is the instrumental contribution to the linewidth (dominated by the effects of imperfect decoupling and field inhomogeneity), so the analysis was performed for upper and lower experimental linewidth limits of 0.3 and 2 Hz respectively, as shown in Fig. S5, with the results listed in Table S1. For full details see the accompanying Mathematica notebook.

500 MHz selective inversion recovery (Hoffman-Forsén) experiments were performed on a 500 MHz Varian VNMRS spectrometer at nominal temperatures from 25 to 50 °C. Analysis in Mathematica used analytical solutions of the Bloch equations for two-site exchange assuming equal longitudinal relaxation rates for the two exchanging methyl resonances. All data for a given temperature were fitted simultaneously; for full details see the accompanying Mathematica notebook. Fitted recovery curves are shown in Fig. S6, and results are summarised in Table S2.

TRUE experiments were performed on a 400 MHz Varian Inova spectrometer at nominal temperatures from 25 to 50 °C. Rate constants were determined using the expressions

$$k = 1/T_2^{CH3} - 1/T_1^{CH3} - 1/T_1^{CH2}$$

for the methyl resonance and

$$k = 1/T_2^{CH2} - 1/T_1^{CH2} - 1.5/T_1^{CH3}$$

for the methylene. Experimental results are summarised in Tables S3 and S4.

Good agreement was obtained between exchange rate constants obtained from the bandshape, Hoffman-Forsén and methyl group TRUE experiments, as shown in the Arrhenius plot of Fig. S7. The methylene TRUE data showed the presence of an extra transverse relaxation contribution of ca. 0.7 s^{-1}, attributable to unresolved long-range coupling. When this contribution is subtracted, excellent agreement is obtained between all measurements (Fig. S8).

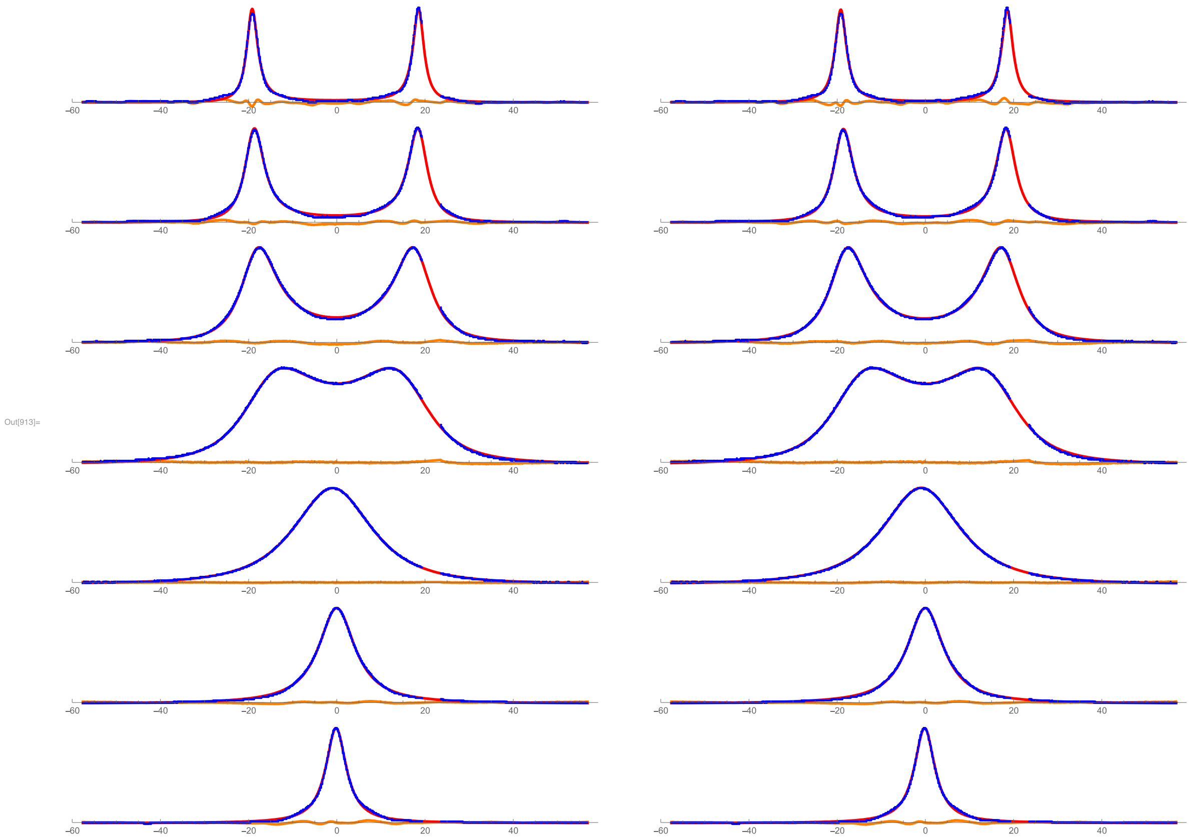

Fig. S5 Methyl region of methylene-decoupled ^{1}H NMR spectra of *N*,*N*-diethylacetamide in DMSO-d_6, with the frequency scale in Hz centred on the average chemical shift. Experimental data, fitted curve and residuals are shown as blue, red, and orange respectively, with fits using estimated instrumental linewidths of 0.3 Hz (left column) and 2 Hz (right column). An impurity peak at +22 Hz was excised from the experimental spectra before fitting. The sample temperature was regulated at nominal temperatures of 50, 60, 70, 80, 90, 100, and 110 °C, respectively, from top to bottom.

Table S1 Results of bandshape analysis of variable temperature ^{1}H DEA spectra. Estimated remaining uncertainty in rate constants ±10%.

**temperature / °C**	**exchange rate constant assuming 0.3 Hz instrumental linewidth / s^{-1}**	**exchange rate constant assuming 2 Hz instrumental linewidth / s^{-1}**
110	413	474
100	237	246
90	128	128
80	63	61
70	30.5	28.1
60	15.4	12.9
50	8.1	5.6

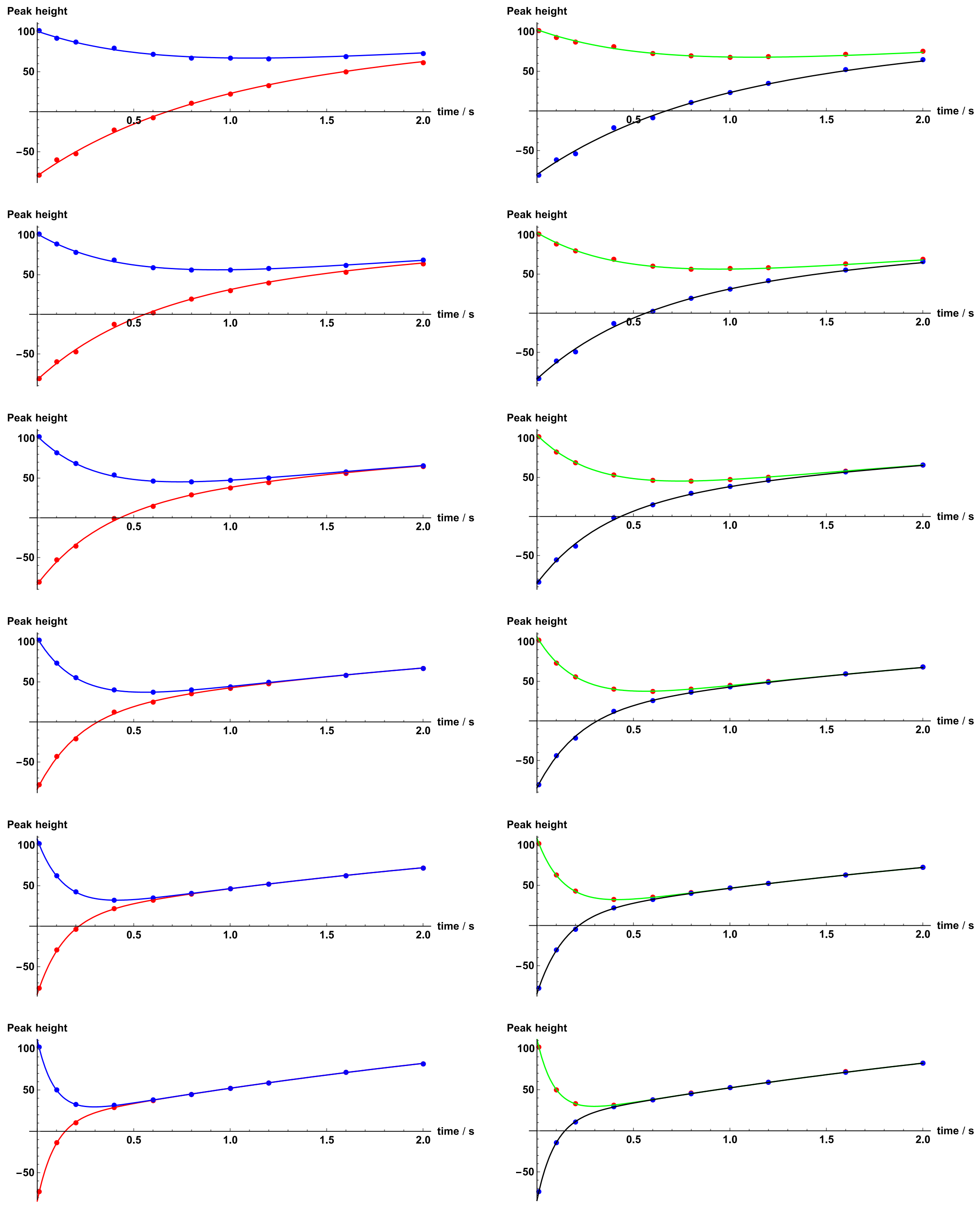

Fig. S6 Experimental methyl signal peak heights after selective inversion of the less shielded (left column) and more shielded (right column) methyl group. The sample temperature was regulated at 25, 30, 35, 40, 45, and 50 °C, respectively, from top to bottom.

Table S2 Results of Hoffmann-Forsén selective inversion recovery analysis of the methyl protons. Errors quoted are twice the standard error estimated in the fitting process.

**temperature / °C**	**exchange rate constant / s^{-1}**
50	5.41 ± 0.06
45	3.46 ± 0.05
40	2.17 ± 0.06
35	1.33 ± 0.05
30	0.79 ± 0.06
25	0.50 ± 0.06

Table S3 Results of relaxation measurements. Errors quoted are twice the standard error estimated in the fitting process.

**temperature / °C**	**T_1 / s**		**T_2 from TRUE / s**		
	CH_3	CH_2	less shielded CH_3	more shielded CH_3	average from overlapping CH_2 protons
50	3.0 ± 0.01	2.8 ± 0.01	0.18 ± 0.01	0.18 ± 0.01	0.15 ± 0.02
45	2.8 ± 0.01	2.7 ± 0.01	0.25 ± 0.01	0.25 ± 0.01	0.21 ± 0.02
40	2.7 ± 0.01	2.4 ± 0.01	0.35 ± 0.01	0.36 ± 0.01	0.27 ± 0.02
35	2.5 ± 0.01	2.2 ± 0.01	0.48 ± 0.01	0.49 ± 0.01	0.33 ± 0.01
30	2.3 ± 0.01	2.1 ± 0.01	0.62 ± 0.01	0.64 ± 0.01	0.38 ± 0.02
25	2.1 ± 0.01	1.9 ± 0.01	0.73 ± 0.02	0.75 ± 0.01	0.43 ± 0.02

Table S4 Exchange rate constants *k* calculated from the relaxation measurements in the slow exchange limit of Table S3 using the equation $k = 1/T_2 - 1/T_1 - \Sigma_i\ 1/(2T_1^i)$, where T_2 and T_1 are the TRUE T_2 and the spin-lattice relaxation time respectively of the resonance of interest, and the summation is over the spin-lattice relaxation rates of all the protons i to which that resonance has resolved couplings. Errors quoted are twice the result of propagating the standard errors estimated in the fitting processes.

**temperature / °C**	**average methyl exchange rate constant / s^{-1}**	**average methylene exchange rate constant / s^{-1}**
50	4.9 ± 0.5	5.8 ± 1.0
45	3.3 ± 0.2	3.9 ± 0.7
40	2.0 ± 0.1	2.7 ± 0.4
35	1.27 ± 0.04	2.0 ± 0.4
30	0.68 ± 0.03	1.5 ± 0.4
25	0.35 ± 0.02	1.1 ± 0.3

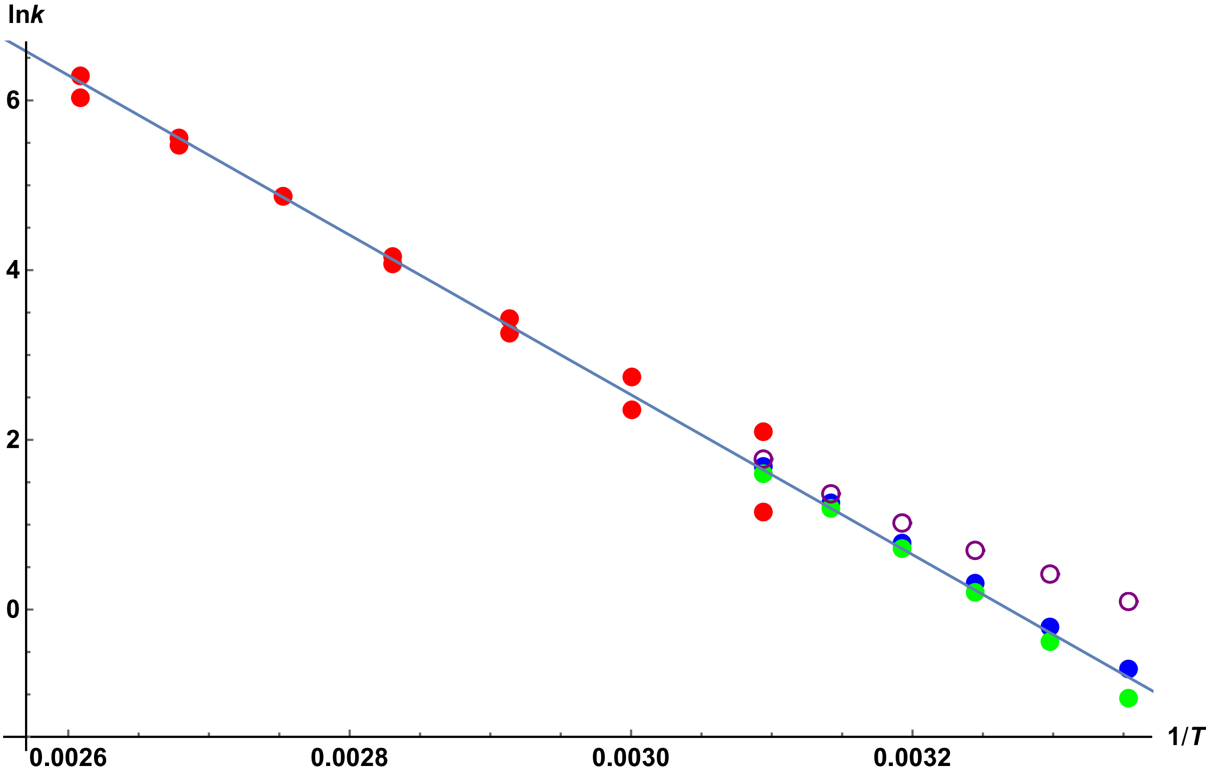

Fig. S7 Arrhenius plot summarising the results of bandshape analysis (red dots), selective inversion recovery (blue dots), and TRUE experiments on methyl (green dots) and methylene (open purple circles) resonances, for the measurement of amide rotation rate. Bandshape analysis was performed for estimated lower and upper limits of extraneous instrumental line broadening of 0.3 Hz and 2 Hz. The methylene TRUE data, which are distorted by the effects of an extra transverse relaxation contribution from unresolved couplings, were omitted from linear regression, which gave an estimated activation energy of 78.3 ± 1.4 kJ mol^{-1}.

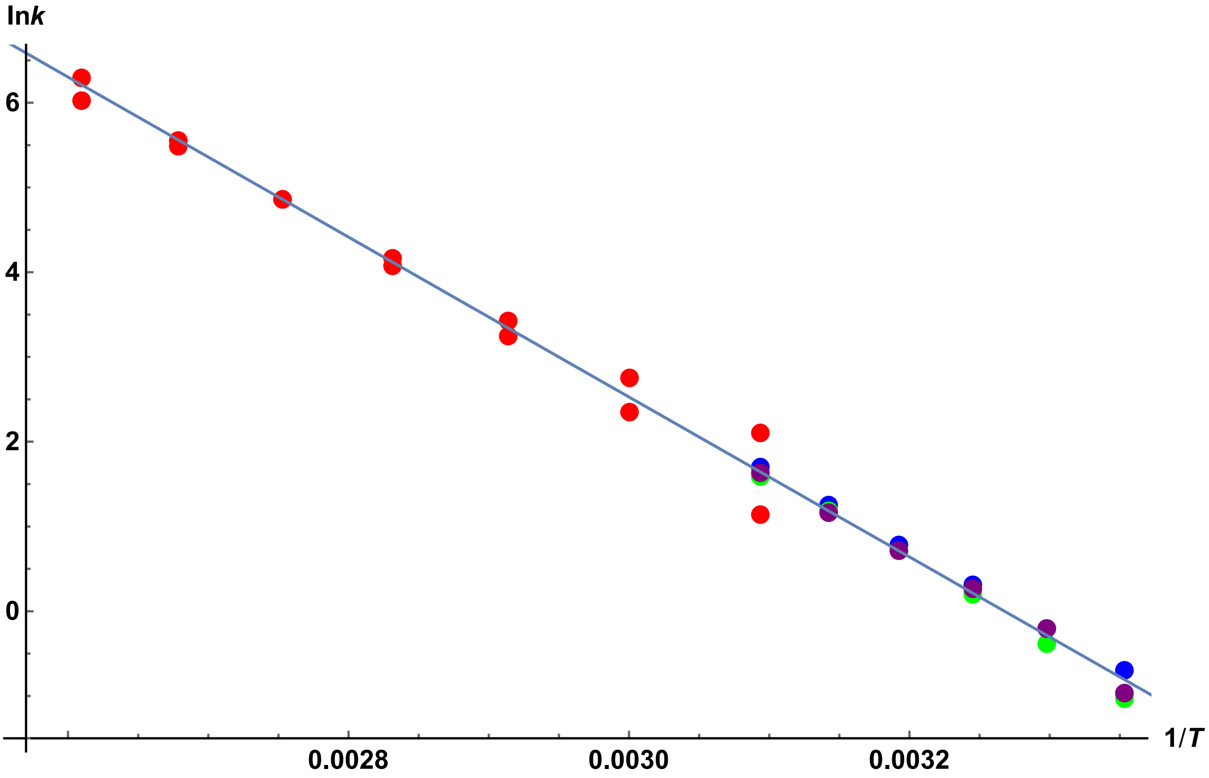

Fig. S8 Arrhenius plot as in Fig. S5, but with an empirical correction of 0.7 s^{-1} applied to the methylene TRUE data to allow for the effects of unresolved couplings. Linear regression of all the data gave an estimated activation energy of 78.5 ± 1.2 kJ mol^{-1}.

4. Study of conformational exchange in $[Au_2(\mu\text{-xantphos})_2](NO_3)_2$ complex

CPMG, TRUE and inversion recovery experiments were performed on a sample of $[Au_2(\mu\text{-xantphos})_2](NO_3)_2$ (PAuP) in CD_2Cl_2 as described in section 3 above and in the main text. Fitted variable temperature 1D ^1H NMR spectra and results of Hoffman-Forsén selective inversion experiments are shown in Figs. S10 and S11 respectively, and results are summarised in Tables S5 to S10.

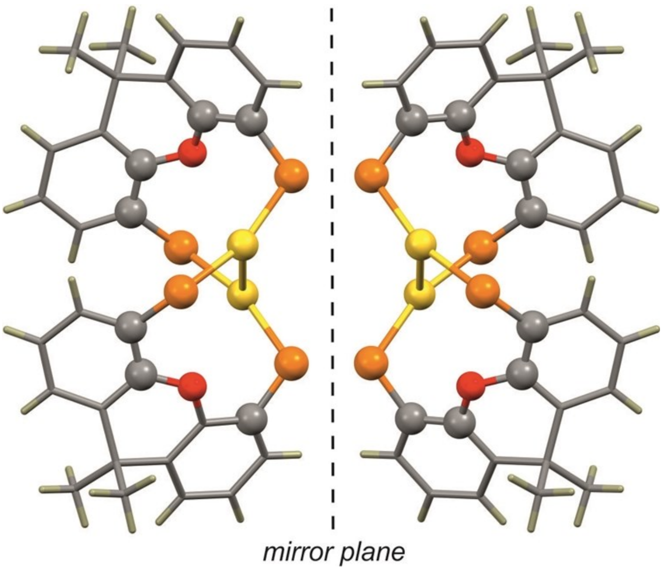

Fig. S9 Capped stick representation of mirror-image conformers of $[Au_2(\mu\text{-xantphos})_2]^{2+}$ cation, wherein the helically folded (figure-eight) skeleton is shown in a ball-and-stick style. The phenyl groups have been omitted for clarity. Colour scheme: gold, yellow; phosphorous, orange; carbon, grey; oxygen, red; hydrogen, light brown.

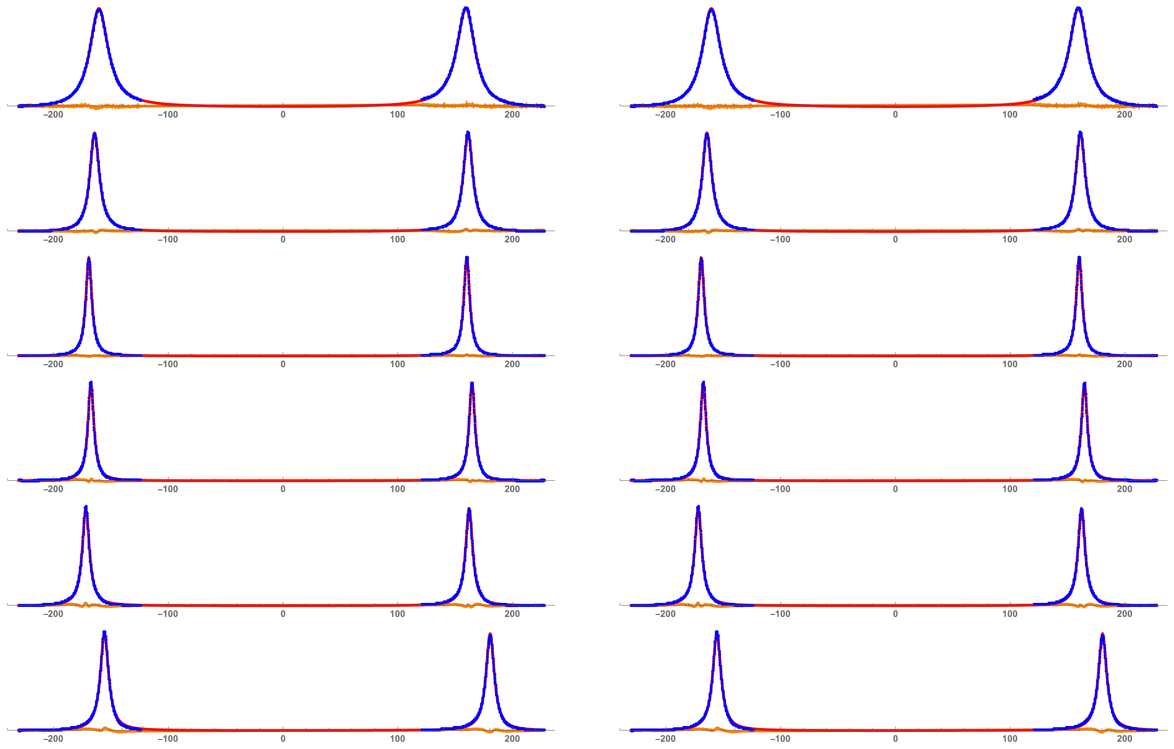

Fig. S10 Methyl region of the variable temperature ^{1}H NMR spectrum of $[Au_2(\mu\text{-xantphos})_2](NO_3)_2$ in CD_2Cl_2, with the frequency scale in Hz centred on the average chemical shift. Experimental data, fitted curve and residuals are shown as blue, red, and orange respectively, with fits using estimated instrumental linewidths of 0.3 Hz (left column) and 2 Hz (right column). The sample temperature was regulated at nominal temperatures of -10, -20, -30, -40, -50, and -60 °C, respectively, from top to bottom.

Table S5 Results of bandshape analysis of variable temperature ^{1}H NMR PAuP spectra. Estimated remaining uncertainty in apparent rate constants ±10%. No correction made for unresolved long-range proton couplings.

**temperature / °C**	**exchange rate constant assuming 0.3 Hz instrumental linewidth / s^{-1}**	**exchange rate constant assuming 2 Hz instrumental linewidth / s^{-1}**
-10	51	45
-20	21	15.6
-30	8.6	3.3
-40	6.9	1.5
-50	8.2	2.8
-60	8.1	2.8

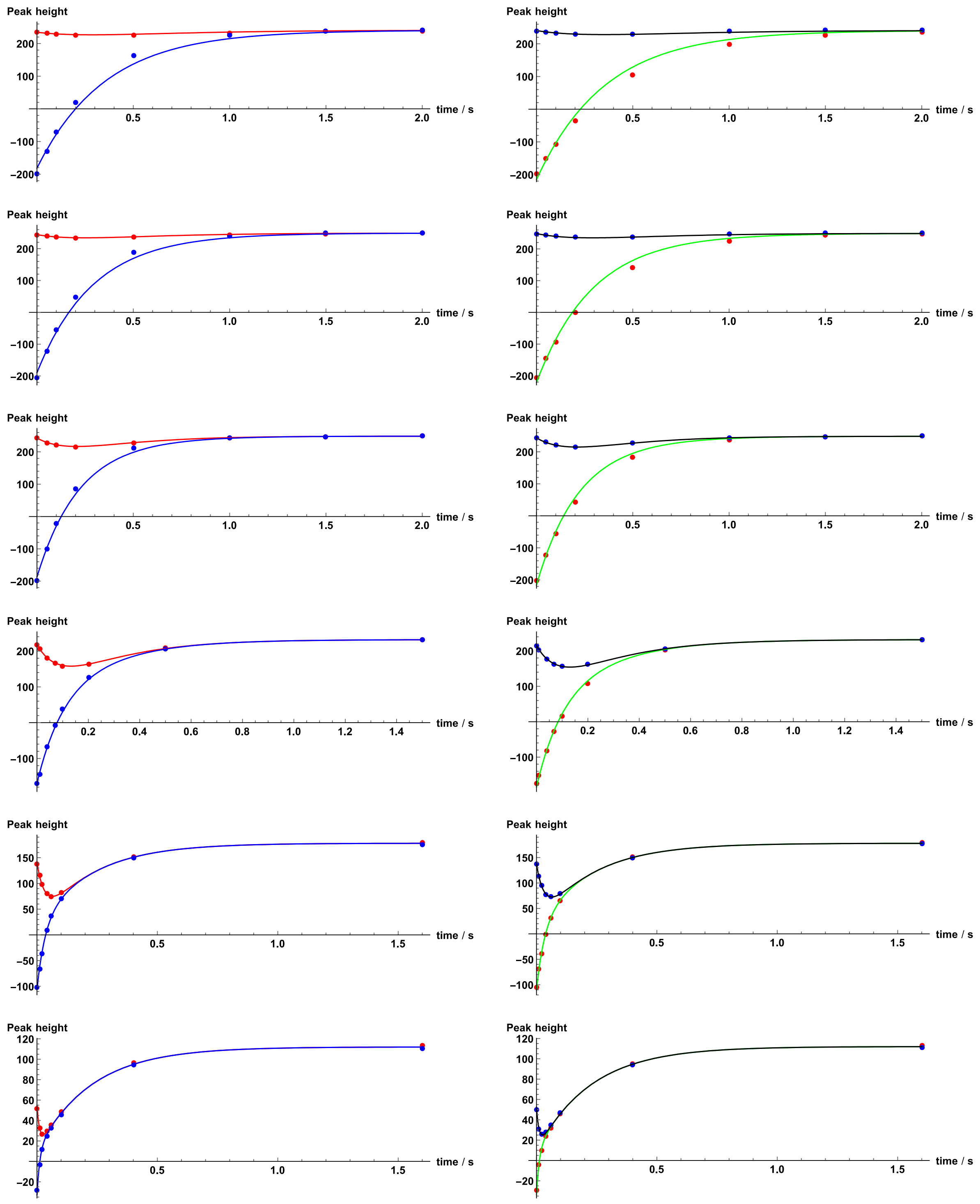

Fig. S11 Experimental methyl signal peak heights after inversion of the equatorial methyl resonance (left) and axial resonance (right), with fits to the analytical solutions of the Bloch-McConnell equations assuming indistinguishable spin-lattice relaxation times. The sample temperature was regulated at nominal temperatures of -60, -50, -40, -30, -20, and -10 °C, from top to bottom.

Table S6 Results of selective inversion recovery analysis of the methyl protons. Uncertainties indicated are twice the standard error estimated in the fitting process.

**temperature / °C**	**exchange rate / s^{-1}**
-10	40.8 ± 2.2
-20	12.9 ± 0.3
-30	3.40 ± 0.04
-40	0.84 ± 0.01
-50	0.28 ± 0.04
-60	0.22 ± 0.03

Table S7 Exchange rates calculated from relaxation measurements

**temperature / °C**	**equatorial methyl exchange rate / s^{-1}**	**axial methyl exchange rate / s^{-1}**	**aromatic proton X3 exchange rate / s^{-1}**
-10	49.5 ± 3.5	49.5 ± 3.5	
-20	18.7 ± 0.8	18.4 ± 0.8	22.7 ± 0.7
-30	6.2 ± 0.3	5.5 ± 0.3	6.4 ± 0.2
-40	2.0 ± 0.2	1.4 ± 0.2	1.5 ± 0.2
-50	1.0 ± 0.3	0.5 ± 0.3	0.34 ± 0.14
-60	0.6 ± 0.5	0.0 ± 0.5	0.04 ± 0.14

Table S8 Relaxation data used for estimating exchange rates for the methyl groups

**temperature / °C**	**T_1 / s**		**T_2 from CPMG / s**		**T_2 from TRUE / s**	
	CH_3 (eq)	CH_3 (ax)	CH_3 (eq)	CH_3 (ax)	CH_3 (eq)	CH_3 (ax)
-10	0.22 ± 0.01	0.22 ± 0.01	0.107 ± 0.001	0.107 ± 0.001	0.017 ± 0.001	0.017 ± 0.001
-20	0.22 ± 0.01	0.23 ± 0.01	0.110 ± 0.001	0.107 ± 0.001	0.036 ± 0.001	0.036 ± 0.001
-30	0.23 ± 0.01	0.25 ± 0.01	0.101 ± 0.001	0.094 ± 0.001	0.062 ± 0.001	0.062 ± 0.001
-40	0.25 ± 0.01	0.31 ± 0.01	0.087 ± 0.001	0.080 ± 0.001	0.074 ± 0.001	0.072 ± 0.001
-50	0.31 ± 0.02	0.40 ± 0.01	0.072 ± 0.001	0.066 ± 0.001	0.067 ± 0.001	0.064 ± 0.001
-60	0.41 ± 0.03	0.52 ± 0.02	0.057 ± 0.001	0.052 ± 0.001	0.055 ± 0.001	0.052 ± 0.001

Table S9 Relaxation data (T_1) used for estimating exchange rates for the aromatic proton (X_3)

temperature / °C	T_1 / s				
	X8	X1	X2	X3	^{31}P
-20	0.83 ± 0.01	0.84 ± 0.01	1.29 ± 0.01	1.62 ± 0.01	2.1 ± 0.1
-30	0.81 ± 0.01	0.80 ± 0.01	1.43 ± 0.01	1.80 ± 0.01	2.1 ± 0.1
-40	0.85 ± 0.01	0.84 ± 0.01	1.59 ± 0.01	2.03 ± 0.01	2.0 ± 0.1
-50	0.92 ± 0.01	0.89 ± 0.01	1.73 ± 0.01	2.23 ± 0.01	2.2 ± 0.1
-60	1.01 ± 0.01	0.98 ± 0.01	1.84 ± 0.01	2.41 ± 0.01	2.5 ± 0.1

Table S10 Relaxation data (T_2) used for estimating exchange rates for the aromatic proton (X_3)

temperature / °C	T_2 from CPMG / s	T_2 from TRUE / s
	proton X3	
-20	0.434 ± 0.004	0.039 ± 0.001
-30	0.467 ± 0.004	0.110 ± 0.001
-40	0.430 ± 0.004	0.228 ± 0.004
-50	0.370 ± 0.003	0.281 ± 0.005
-60	0.293 ± 0.003	0.255 ± 0.004

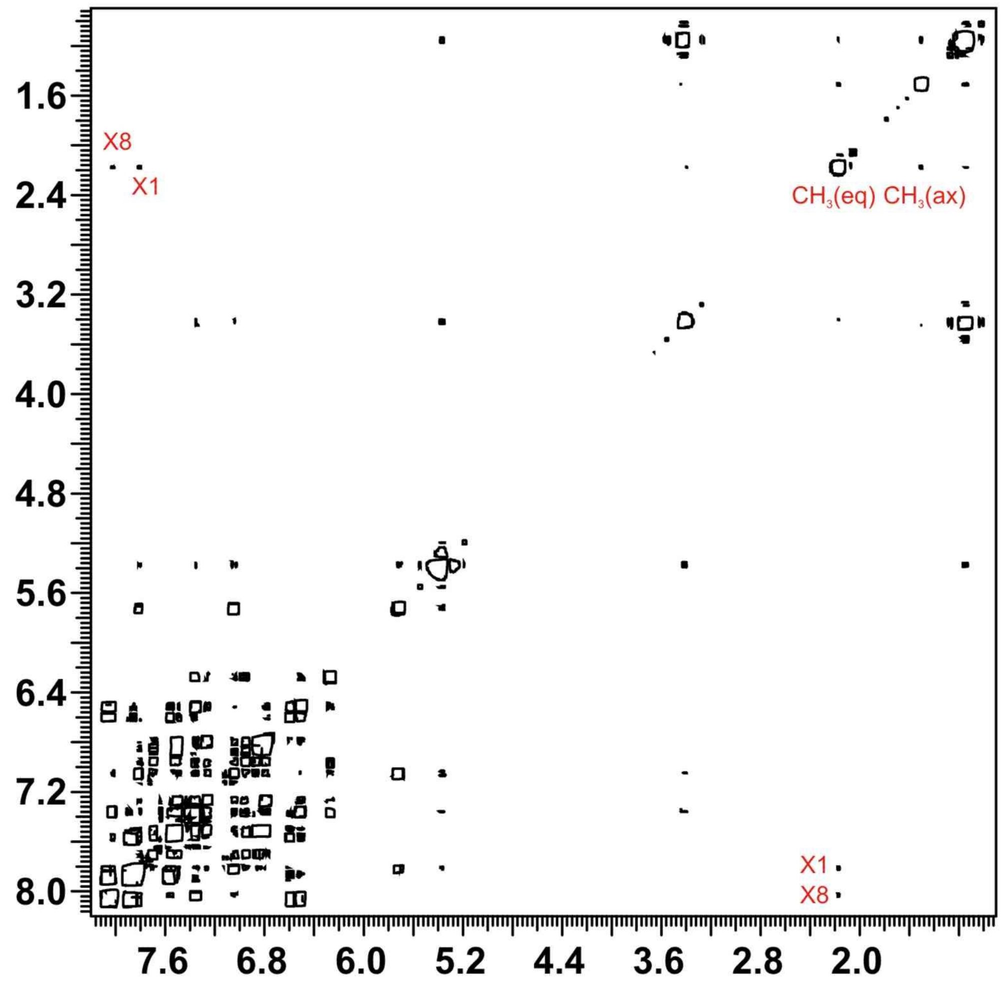

Fig. S12 gCOSY spectrum of $[Au_2(\mu\text{-xantphos})_2](NO3)_2$ in CD_2Cl_2 at -45°C. The cross-peaks between the equatorial and axial methyl protons, and the equatorial methyl and closest aromatic protons of the xanthene ring (X1 and X8) are due to long-range proton *J*-coupling which is not resolved in the methyl proton signals but contribute to their linewidth significantly. The experiment was optimised to observe such long-range couplings using a 160 ms fixed evolution time prior to t_1. 2 scans and 108 increments were collected using a spectral width of 5 kHz. Sinebell apodisation, zero filling to 4096 in both dimensions and symmetrisation were applied in magnitude mode 2D FT processing.

5. Pulse programs for Bruker spectrometers

```
;T2 experiment using simple spin echo with Zangger-Sterk element
;
; Developed by NMR Group
; School of Chemistry, University of Manchester, United Kingdom
; Dec 2017, Peter Kiraly
;
;$CLASS=HighRes
;$DIM=2D
;$TYPE=
;$SUBTYPE=
;$COMMENT=


#include <Avance.incl>
#include <Delay.incl>
#include <Grad.incl>
#include <De.incl>

define list<delay> kp_list = <$VDLIST>

"p2=p1*2"
"d11=30m+1s/(cnst50)-1s/(cnst50)"
"d11=30m"
"d17=1m"

"acqt0=0.0"
baseopt_echo


1 ze

2 d11
  50u BLKGRAMP
  50u LOCKH_OFF
  d1 pl1:f1
     "d17=kp_list/2"
  50u LOCKH_ON
```

```
  50u UNBLKGRAMP

  p1 ph1
  d17
  p16:gp16
      d16
      4u gron0
      4u
      (p12:sp12 ph2):f1
      4u groff
      4u

  p16:gp16
  d16
  d17

  go=2 ph31
  ;d11 wr #0 if #0 kp_list.inc
  ;lo to 1 times td1
  d11 mc #0 to 2 F1QF(kp_list.inc)

exit


 ph1 = 0 0 0 0  2 2 2 2   1 1 1 1 3 3 3 3
 ph2 = 0 1 2 3  0 1 2 3   0 1 2 3 0 1 2 3
ph31 = 2 0 2 0  0 2 0 2   1 3 1 3 3 1 3 1



;cnst50: bandwidth of the selective rsnob pulse [Hz]
;sp12(p12):wvm:kp_soft:f1 rsnob(cnst50 Hz; NPOINTS=1000)


;pl1 : f1 channel - power level for pulse (default)
;p1 : f1 channel -  90 degree high power pulse
;p2 : f1 channel - 180 degree high power pulse
;d1 : relaxation delay; 1-5 * T1
;d11: delay for disk I/O [30m]
;d16: gradient stabilisation delay [1m]
;p16: gradient pulse [1m]
```

```
;gpz16: gradient pulse power [29-53%]
;gpnam16: gradient pulse shape
;
;vd : variable delay, taken from vd-list
;ns: 1*n (recommended 4)
;ds: 4
;td1: number of experiments = number of delays in vd-list
;FnMODE: QF

;define VDLIST

;this pulse program produces a ser-file (PARMOD = 2D)
```

6. Pulse programs for Varian/Agilent spectrometers

```
// broadband T2 experiment using a simple spin echo with Zangger=Sterk
element


/*----------------------
Developed By NMR Group
School of Chemistry
University of Manchester
United Kingdom
Dec 2017
----------------------*/
```

```
#include <standard.h>

static int ph1[16] = {0,0,0,0, 2,2,2,2, 1,1,1,1, 3,3,3,3},  // v1: first
pulse
           ph2[16] = {0,1,2,3, 0,1,2,3, 0,1,2,3, 0,1,2,3},  // v2:
refocusing pulse
           ph5[16] = {2,0,2,0, 0,2,0,2, 1,3,1,3, 3,1,3,1};  // oph:
receiver

pulsesequence()
{
double      bigtau = getval("bigtau"),

      gstab = getval("gstab"),
      gt1 = getval("gt1"),
      gzlvl1 = getval("gzlvl1"),
      gzlvl7 = getval("gzlvl7"),
      kp_pfgtc = getval("kp_pfgtc"),

      pw180_a = getval("pw180_a"),
      pwr180_a = getval("pwr180_a");

char  shp_a[MAXSTR], lkgate_flg[MAXSTR];
      getstr("shp_a",shp_a);
      getstr("lkgate_flg",lkgate_flg);


      settable(t1,16,ph1);
      settable(t2,16,ph2);
      settable(t5,16,ph5);

      getelem(t1, ct, v1);
      getelem(t2, ct, v2);
      getelem(t5, ct, oph);


status(A);
      obspower(tpwr);
      txphase(zero);
      obsoffset(tof);
      delay(50.0e-3);
```

```
if (lkgate_flg[0] == 'y')  lk_sample();
      delay(d1);
if (lkgate_flg[0] == 'y')  lk_hold();
      delay(0.1);

status(B);
      rgpulse(pw,v1,rof1,0.0);
      obspower(pwr180_a);
      delay(bigtau/2.0 -50.0e-9);
      if (gt1>0.0)
      {
      zgradpulse(gzlvl1,gt1);
      delay(gstab);
      }
      rgradient('z',gzlvl7);
      delay(kp_pfgtc);
      shaped_pulse(shp_a,pw180_a,v2,rof1,rof1);
      rgradient('z',0.0);
      delay(kp_pfgtc);
      if (gt1>0.0)
      {
      zgradpulse(gzlvl1,gt1);
      delay(gstab);
      }
      delay(bigtau/2.0);
setacqmode(WACQ|NZ);
      obsblank();
      delay(rof2);
      startacq(alfa);
status(C);
      acquire(np,1.0/sw);
      recoff();
      endacq();
if (lkgate_flg[0] == 'y')  lk_sample();
}
```

7. Matlab source code for numerical simulations with the spinach program libraries

Two Matlab functions were written, one to define the spin system (kp_sample_T2exps_01.m) and one to define the pulse sequences (kp_T2exps_01.m). Their content is given below, respectively.

```
% sample, spin system definition file for T2 experiments
%
% Feb 2019
% Peter Kiraly
% NMR Methodology Group
% University of Manchester
%

function
[fid]=kp_sample_T2exps_01(subdir,kp_approx,T1a,T1x,T2a,T2x,jhh
,tau,pw)

switch ispc
    case 0

[KP_PATH_OUTPUT]=['/media/hamster/PK/2018/spinach/kp_results/T
2/' subdir '/'];
    case 1
    [KP_PATH_OUTPUT]=['C:/space/spinach/kp_results/T2/' subdir
'/'];
end

if ~exist(KP_PATH_OUTPUT,'dir')
```

```
    mkdir(KP_PATH_OUTPUT);
end

% copy the spin system definition matlab file to the results
directory
funtion_str = [mfilename('fullpath') '.m'];
copyfile(funtion_str,KP_PATH_OUTPUT);



BF1=500;
gamma_1H=267.5222005/(2*pi);
sys.magnet=BF1/gamma_1H;

% redirect spinach log to file
%sys.output=[ KP_PATH_OUTPUT +'matlab_logfile.txt'];

% speeding up calculations by bypassing the output to console
sys.output='hush';

% Spin system and relaxation model
        sys.isotopes={'1H','1H'};
        inter.zeeman.scalar={0.6 -0.4};

        inter.coupling.scalar=cell(2,2);
        inter.coupling.scalar{1,2}=jhh;

        inter.relaxation={'t1_t2'};
        inter.rlx_keep='secular';
        inter.equilibrium='zero';
        inter.r1_rates=[1/T1a, 1/T1x];
        inter.r2_rates=[1/T2a, 1/T2x];
```

```
bas.formalism='sphten-liouv';
bas.approximation='IK-2';
bas.connectivity='scalar_couplings';
bas.space_level=1;

spin_system=create(sys,inter);
spin_system=basis(spin_system,bas);


% Sequence parameters

parameters.spins={'1H'};
parameters.axis_units='Hz';
parameters.resultsdir = KP_PATH_OUTPUT;

parameters.sw=5000;
parameters.np=4096*8;

parameters.pw=pw; % 90 pulse duration [us]

parameters.tau=tau;
switch tau
     case 0.00005

parameters.cycles=[64,128,256,512,1024,2048,4096,8192];
     case 0.0001
        parameters.cycles=[32,64,128,256,512,1024,2048,4096];
     case 0.0002
        parameters.cycles=[16,32,64,128,256,512,1024,2048];
     case 0.0004
        parameters.cycles=[8,16,32,64,128,256,512,1024];
     case 0.0008
        parameters.cycles=[4,8,16,32,64,128,256,512];
     otherwise
```

```
        parameters.cycles=[16,32,64,128,256,512,1024,2048];
end
parameters.bigtau=parameters.cycles*parameters.tau*2;

parameters.samplename='AX';


% write text file with input argument values
file_input = fopen([ parameters.resultsdir 'kp_input.txt'
],'w');

fprintf(file_input,'[fid]=%s(''%s'',%d,%.3f,%.3f,%.3f,%.3f,%.3
f,%.6f,%.4f);\n',mfilename,subdir,kp_approx,T1a,T1x,T2a,T2x,jh
h,tau,pw);
 fprintf(file_input,'\n');
 fprintf(file_input,'function name: %s \n',funtion_str);
 fprintf(file_input,'arg-1[directory]: %s \n',subdir);
 fprintf(file_input,'arg-2[weak/strong coupling]:
%d\n',kp_approx);
 fprintf(file_input,'arg-3[T1a]: %.6f\n',T1a);
 fprintf(file_input,'arg-4[T1x]: %.6f\n',T1x);
 fprintf(file_input,'arg-5[T2a]: %.6f\n',T2a);
 fprintf(file_input,'arg-6[T2x]: %.6f\n',T2x);
 fprintf(file_input,'arg-7[Jax]: %.3f\n',jhh);
 fprintf(file_input,'arg-8[echo time]: %.6f\n',tau);
 fprintf(file_input,'arg-9[pw90]: %.3f\n',pw);
fclose(file_input);


% Simulation
if (kp_approx>0)
    [fid]=liquid(spin_system,@kp_T2exps_01,parameters,'nmr');
else
    [fid]=liquid(spin_system,@kp_T2exps_01,parameters,'wnmr');
```

```
end

end

% pulse sequence file for T2 experiments
%
% Feb 2019
% Peter Kiraly
% NMR Methodology Group
% University of Manchester
%
%   _01 version cleaned-up for publication
%       experiments include: CPMG, PROJECT, ASR using
ideal/real pulses


function [fid]=kp_T2exps_01(spin_system,parameters,H,R,K)

tic

switch ispc
    case 0
    [KP_PATH_TOOLS]='/media/hamster/PK/2018/spinach/kp_tools';
    case 1
    [KP_PATH_TOOLS]='C:/space/spinach/kp_tools';
    %[KP_PATH_TOOLS]='H:/PK/2018/spinach/kp_tools';
end


%write a log file with time
file_header_log = fopen([ parameters.resultsdir 'kp_time.log'
],'w');
 fprintf(file_header_log,'Matlab file: %s \n',mfilename);
fclose(file_header_log);
```

```
bigtau=parameters.bigtau;

%write header file
file_header = fopen([ parameters.resultsdir
'kp_spinach_parameters.txt' ],'w');
 fprintf(file_header,'np %d \n',2*parameters.np);
 fprintf(file_header,'sw %d \n',parameters.sw);
  for k=1:length(bigtau)
     k_str = sprintf('%d',k);
     fprintf(file_header,['bigtau %.6f,' k_str
'\n'],bigtau(k));
 end
 fprintf(file_header,'tau %.6f \n',parameters.tau);
 fprintf(file_header,'pw %.3f \n',parameters.pw);
 fprintf(file_header,'phase %d \n',0);
%  fprintf(file_header,'kp_cycles %d \n',parameters.cycles);
 fprintf(file_header,'seqfil %s \n','kp_T2_pub');
 fprintf(file_header,'pslabel %s \n',mfilename);

fclose(file_header);



% copy the pulse sequence matlab file to the results directory
funtion_str = [mfilename('fullpath') '.m'];
copyfile(funtion_str,parameters.resultsdir);

% Compose Liouvillian
L=H+1i*R+1i*K; clear('H','R','K');

% Initial state
rho=state(spin_system,'Lz','1H');

% Detection state
```

```
coil=state(spin_system,'L+','1H');

% Pulse operators for
% instant, ideal and broadband proton pulses
 Lp=operator(spin_system,'L+','1H');
 Lx=(Lp+Lp')/2; Ly=(Lp-Lp')/2i;
% instant, ideal and selective proton pulses for 2 spins
 Lp1=operator(spin_system,'L+',1);
 Lx1=(Lp1+Lp1')/2; Ly1=(Lp1-Lp1')/2i;
 Lp2=operator(spin_system,'L+',2);
 Lx2=(Lp2+Lp2')/2; Ly2=(Lp2-Lp2')/2i;

% realistic broadband proton pulses
  pw=parameters.pw*1.0e-6;    % 90 pulse duration [s]
  tpwr=2.0*pi/(4.0*pw); % 2PI*field strength

% realistic soft pulse using RSNOB with 100Hz effective
bandwidth
pw_BASHD = 18500; pwr_BASHD=126.0;
[BASHD_shape(:,1),BASHD_shape(:,2)]=read_wave([KP_PATH_TOOLS
'/kp_rsnob_np400'],400);

L_samp = 1.8; % length of the sample in cm
Gz7 = 1600/( L_samp); % ZS gradient amplitude
slices=1600; % parameters.slices;
z=linspace(-L_samp/2,L_samp/2,slices);
%rho_tot_ZS=0;


% parameters
sw=parameters.sw;
np=parameters.np;

tau=parameters.tau;
```

```
cycles=parameters.cycles;

%write a log file with time
file_header_log = fopen([ parameters.resultsdir 'kp_time.log'
],'w');
kp_time=toc;
fprintf(file_header_log,'before experiment: %8.4f sec
\n',kp_time);



fid=cell(1,20);

kp_expnum=1;
%%%%%%%%%%%%%%%%%%%%%%%%%%%%%%%%%%%%%%%%%%%%%%%%%%%%%%%%%%%
% SE: hard spin echo using ideal pulses

    rho1_SE=cell(1,length(bigtau));
    parfor k=1:length(bigtau)
        rho1_SE{k}=step(spin_system,Lx,rho,pi/2);

rho1_SE{k}=evolution(spin_system,L,[],rho1_SE{k},tau*cycles(k)
,1,'final');
        rho1_SE{k}=step(spin_system,Ly,rho1_SE{k},pi);

rho1_SE{k}=evolution(spin_system,L,[],rho1_SE{k},tau*cycles(k)
,1,'final');
    end

    for k=1:length(bigtau)

fid{1,kp_expnum}(:,k)=evolution(spin_system,L,coil,rho1_SE{k},
1.0/sw,np-1,'observable');
    end
```

```
    % write FIDs

kp_write_resultsV(fid{1,kp_expnum},'V2',[parameters.resultsdir
'iSE_']);

kp_time=toc;
fprintf(file_header_log,'end of spin-echo (ideal): %8.4f min
\n',kp_time/60);
kp_expnum=kp_expnum+1;
%%%%%%%%%%%%%%%%%%%%%%%%%%%%%%%%%%%%%%%%%%%%%%%%%%%%%%%%%%%

%%%%%%%%%%%%%%%%%%%%%%%%%%%%%%%%%%%%%%%%%%%%%%%%%%%%%%%%%%%%
% CPMG using broadband ideal pulses

    rho1_CPMG=cell(1,length(bigtau));
    parfor k=1:length(bigtau)
        rho1_CPMG{k}=step(spin_system,Lx,rho,pi/2);

        for i=1:cycles(k)

rho1_CPMG{k}=evolution(spin_system,L,[],rho1_CPMG{k},tau,1,'fi
nal');
            rho1_CPMG{k}=step(spin_system,Ly,rho1_CPMG{k},pi);

rho1_CPMG{k}=evolution(spin_system,L,[],rho1_CPMG{k},tau,1,'fi
nal');
        end
    end

    for k=1:length(bigtau)

fid{1,kp_expnum}(:,k)=evolution(spin_system,L,coil,rho1_CPMG{k
},1.0/sw,np-1,'observable');
```

```
    end

    % write FIDs

kp_write_resultsV(fid{1,kp_expnum},'V2',[parameters.resultsdir
'iCPMG_']);

kp_time=toc;
fprintf(file_header_log,'end of CPMG (ideal): %8.4f min
\n',kp_time/60);
kp_expnum=kp_expnum+1;
%%%%%%%%%%%%%%%%%%%%%%%%%%%%%%%%%%%%%%%%%%%%%%%%%%%%%%%%

%%%%%%%%%%%%%%%%%%%%%%%%%%%%%%%%%%%%%%%%%%%%%%%%%%%%%%%%%%
% PROJECT using broadband ideal pulses

    rho1_PR=cell(1,length(bigtau));
    parfor k=1:length(bigtau)
        rho1_PR{k}=step(spin_system,Lx,rho,pi/2);

        for i=1:cycles(k)

rho1_PR{k}=evolution(spin_system,L,[],rho1_PR{k},tau/2.0,1,'fi
nal');
            rho1_PR{k}=step(spin_system,Lx,rho1_PR{k},pi);

rho1_PR{k}=evolution(spin_system,L,[],rho1_PR{k},tau/2.0,1,'fi
nal');
            rho1_PR{k}=step(spin_system,Ly,rho1_PR{k},pi/2);

rho1_PR{k}=evolution(spin_system,L,[],rho1_PR{k},tau/2.0,1,'fi
nal');
            rho1_PR{k}=step(spin_system,Lx,rho1_PR{k},pi);
```

```
rho1_PR{k}=evolution(spin_system,L,[],rho1_PR{k},tau/2.0,1,'fi
nal');

        end
    end

    for k=1:length(bigtau)

fid{1,kp_expnum}(:,k)=evolution(spin_system,L,coil,rho1_PR{k},
1.0/sw,np-1,'observable');
    end

    % write FIDs

kp_write_resultsV(fid{1,kp_expnum},'V2',[parameters.resultsdir
'iPR_']);

kp_time=toc;
fprintf(file_header_log,'end of PROJECT (ideal): %8.4f min
\n',kp_time/60);
kp_expnum=kp_expnum+1;
%%%%%%%%%%%%%%%%%%%%%%%%%%%%%%%%%%%%%%%%%%%%%%%%%%%%%%%%%%%%

%%%%%%%%%%%%%%%%%%%%%%%%%%%%%%%%%%%%%%%%%%%%%%%%%%%%%%%%%%%%%%
% TRUE using ideal soft pulses modelling ZS with broadband
excitation and selective
% refocusing

    rho1_ZS=cell(1,length(bigtau));
    rho1_ZS1=cell(1,length(bigtau));
    rho1_ZS2=cell(1,length(bigtau));
    parfor k=1:length(bigtau)
        rho1_ZS1{k}=step(spin_system,Lx,rho,pi/2);
```

```
        rho1_ZS2{k}=step(spin_system,Lx,rho,pi/2);

rho1_ZS1{k}=evolution(spin_system,L,[],rho1_ZS1{k},tau*cycles(
k),1,'final');

rho1_ZS2{k}=evolution(spin_system,L,[],rho1_ZS2{k},tau*cycles(
k),1,'final');

rho1_ZS1{k}=coherence(spin_system,rho1_ZS1{k},{{'1H',[0,-
1]}});

rho1_ZS2{k}=coherence(spin_system,rho1_ZS2{k},{{'1H',[0,-
1]}});
        rho1_ZS1{k}=step(spin_system,Ly1,rho1_ZS1{k},pi);
        rho1_ZS2{k}=step(spin_system,Ly2,rho1_ZS2{k},pi);

rho1_ZS1{k}=evolution(spin_system,L,[],rho1_ZS1{k},tau*cycles(
k),1,'final');

rho1_ZS2{k}=evolution(spin_system,L,[],rho1_ZS2{k},tau*cycles(
k),1,'final');

rho1_ZS1{k}=coherence(spin_system,rho1_ZS1{k},{{'1H',[0,+1]}})
;

rho1_ZS2{k}=coherence(spin_system,rho1_ZS2{k},{{'1H',[0,+1]}})
;
    end

    for k=1:length(bigtau)

fid{1,kp_expnum}(:,k)=evolution(spin_system,L,coil,rho1_ZS1{k}
,1.0/sw,np-1,'observable');
```

```
fid{1,kp_expnum+1}(:,k)=evolution(spin_system,L,coil,rho1_ZS2{
k},1.0/sw,np-1,'observable');

fid{1,kp_expnum+2}(:,k)=fid{1,kp_expnum}(:,k)+fid{1,kp_expnum+
1}(:,k);
    end

    % write FIDs

kp_write_resultsV(fid{1,kp_expnum},'V2',[parameters.resultsdir
'iZS1_']);

kp_write_resultsV(fid{1,kp_expnum+1},'V2',[parameters.resultsd
ir 'iZS2_']);

kp_write_resultsV(fid{1,kp_expnum+2},'V2',[parameters.resultsd
ir 'iZS_']);

kp_time=toc;
fprintf(file_header_log,'end of ideal ZS (ideal soft pulses):
%8.4f min \n',kp_time/60);
kp_expnum=kp_expnum+3;
%%%%%%%%%%%%%%%%%%%%%%%%%%%%%%%%%%%%%%%%%%%%%%%%%%%%%%%%%%%%



%%%%%%%%%%%%%%%%%%%%%%%%%%%%%%%%%%%%%%%%%%%%%%%%%%%%%%%%%%%%
% ZS using real pulses; single scan; perfect CTP

    rho1_ZS=cell(1,length(bigtau));
    rho2_ZS=cell(1,length(bigtau));
    rho_tot_ZS=cell(1,length(bigtau));
    parfor k=1:length(bigtau)
```

```
rho1_ZS{k}=shaped_pulse(spin_system,L,rho,'1H',0.0,0,tpwr,pw);

rho1_ZS{k}=coherence(spin_system,rho1_ZS{k},{{'1H',[0,-1]}});

rho1_ZS{k}=evolution(spin_system,L,[],rho1_ZS{k},tau*cycles(k)
,1,'final');
        rho_tot_ZS{k}=0;
        for kk=1:length(z)

rho2_ZS{k}=shaped_pulse_gr(spin_system,L,rho1_ZS{k},'1H',0.0,9
0+BASHD_shape(:,2),pwr_BASHD*2*pi*(BASHD_shape(:,1))/100,pw_BA
SHD*1e-6,Gz7,z(kk));
            rho_tot_ZS{k}=rho_tot_ZS{k}+rho2_ZS{k};
        end
        rho_tot_ZS{k}=rho_tot_ZS{k}/length(z);
        rho3_ZS{k}=rho_tot_ZS{k};

rho3_ZS{k}=evolution(spin_system,L,[],rho3_ZS{k},tau*cycles(k)
,1,'final');

rho3_ZS{k}=coherence(spin_system,rho3_ZS{k},{{'1H',[0,+1]}});
    end

    for k=1:length(bigtau)

fid{1,kp_expnum}(:,k)=evolution(spin_system,L,coil,rho3_ZS{k},
1.0/sw,np-1,'observable');
    end

    % write FIDs

kp_write_resultsV(fid{1,kp_expnum},'V2',[parameters.resultsdir
'rZS_']);
```

```
kp_time=toc;
fprintf(file_header_log,'end of ZS (real): %8.4f min
\n',kp_time/60);
kp_expnum=kp_expnum+1;
%%%%%%%%%%%%%%%%%%%%%%%%%%%%%%%%%%%%%%%%%%%%%%%%%%%%%%%%%%%%%



%%%%%%%%%%%%%%%%%%%%%%%%%%%%%%%%%%%%%%%%%%%%%%%%%%%%%%%%%%%%%%%
% CPMG using real pulses; conventional implementation

    rho1_CPMG=cell(1,length(bigtau));
    parfor k=1:length(bigtau)
        rho1_CPMG{k}=step(spin_system,Lx,rho,pi/2);
        for i=1:cycles(k)

rho1_CPMG{k}=evolution(spin_system,L,[],rho1_CPMG{k},tau,1,'fi
nal');

rho1_CPMG{k}=shaped_pulse(spin_system,L,rho1_CPMG{k},'1H',0.0,
90,tpwr,2.0*pw);

rho1_CPMG{k}=evolution(spin_system,L,[],rho1_CPMG{k},tau,1,'fi
nal');
        end
    end

    for k=1:length(bigtau)

fid{1,kp_expnum}(:,k)=evolution(spin_system,L,coil,rho1_CPMG{k
},1.0/sw,np-1,'observable');
    end
```

```
    % write FIDs

kp_write_resultsV(fid{1,kp_expnum},'V2',[parameters.resultsdir
'rCPMG_']);

kp_time=toc;
fprintf(file_header_log,'end of CPMG (real, conv): %8.4f min
\n',kp_time/60);
kp_expnum=kp_expnum+1;
%%%%%%%%%%%%%%%%%%%%%%%%%%%%%%%%%%%%%%%%%%%%%%%%%%%%%%%%%


%%%%%%%%%%%%%%%%%%%%%%%%%%%%%%%%%%%%%%%%%%%%%%%%%%%%%%%%%%%
% CPMG using real pulses; subtracting finite pulse duration

    rho1_CPMG=cell(1,length(bigtau));
    parfor k=1:length(bigtau)

rho1_CPMG{k}=shaped_pulse(spin_system,L,rho,'1H',0.0,0,tpwr,pw
);
        for i=1:cycles(k)

rho1_CPMG{k}=evolution(spin_system,L,[],rho1_CPMG{k},tau-
pw,1,'final');

rho1_CPMG{k}=shaped_pulse(spin_system,L,rho1_CPMG{k},'1H',0.0,
90,tpwr,2.0*pw);

rho1_CPMG{k}=evolution(spin_system,L,[],rho1_CPMG{k},tau-
pw,1,'final');
        end
    end

    for k=1:length(bigtau)
```

```
fid{1,kp_expnum}(:,k)=evolution(spin_system,L,coil,rho1_CPMG{k
},1.0/sw,np-1,'observable');
    end

    % write FIDs

kp_write_resultsV(fid{1,kp_expnum},'V2',[parameters.resultsdir
'rCPMGk_']);

kp_time=toc;
fprintf(file_header_log,'end of CPMG (real, corrected): %8.4f
min \n',kp_time/60);
kp_expnum=kp_expnum+1;
%%%%%%%%%%%%%%%%%%%%%%%%%%%%%%%%%%%%%%%%%%%%%%%%%%%%%%%%%

%%%%%%%%%%%%%%%%%%%%%%%%%%%%%%%%%%%%%%%%%%%%%%%%%%%%%%%%%
%%%%%%%%%%%%%%%%%%%%%%%%%%%%%%%%%%%%%%%%%%%%%%%%%%%%%%%%%
kp_time=toc;
fprintf(file_header_log,'end of experiment: %8.4f min
\n',kp_time/60);

fclose(file_header_log);
end
```
